# ZDHHC15 promotes glioma malignancy and acts as a novel prognostic biomarker for patients with glioma

**DOI:** 10.1186/s12885-023-10883-6

**Published:** 2023-05-09

**Authors:** Zhen-Yuan Liu, Tian Lan, Feng Tang, Yong-Ze He, Jin-Sheng Liu, Jin-Zhou Yang, Xi Chen, Ze-Fen Wang, Zhi-Qiang Li

**Affiliations:** 1 Brain Glioma Center, Department of Neurosurgery, Zhongnan Hospital of Wuhan University, Wuhan, China; 2 Department of Physiology, Wuhan University School of Basic Medical Sciences, Wuhan, China

**Keywords:** Zinc finger DHHC-type palmitoyltransferase 15, Prognosis, Proliferation, Migration, STAT3 signaling pathway, Glioma

## Abstract

**Background:**

Glioma is the most common and aggressive tumor in the adult brain. Recent studies have indicated that Zinc finger DHHC-type palmitoyltransferases (ZDHHCs) play vital roles in regulating the progression of glioma. ZDHHC15, a member of the ZDHHCs family, participates in various physiological activities in the brain. However, the biological functions and related mechanisms of ZDHHC15 in glioma remain poorly understood.

**Methods:**

Data from multiple glioma-associated datasets were used to investigate the expression profiles and potential biological functions of ZDHHC15 in glioma. Expression of ZDHHC15 and its association with clinicopathological characteristics in glioma were validated by quantitative reverse transcription PCR (RT-qPCR) and immunohistochemical experiments. GO enrichment analysis, KEGG analysis, GSEA analysis, CCK-8, EdU, transwell, and western blotting assays were performed to confirm the functions and mechanism of ZDHHC15 in glioma. Moreover, we performed Kaplan-Meier analysis and Cox progression analysis to explore the prognostic significance of ZDHHC15 in glioma patients.

**Results:**

ZDHHC15 expression was significantly up-regulated in glioma and positively associated with malignant phenotypes. Results from the GO and KEGG enrichment analysis revealed that ZDHHC15 was involved in regulating cell cycle and migration. Knockdown of ZDHHC15 inhibited glioma cell proliferation and migration, while overexpression of ZDHHC15 presented opposite effects on glioma cells. Besides, results from GSEA analysis suggested that ZDHHC15 was enriched in STAT3 signaling pathway. Knockdown or overexpression of ZDHHC15 indeed affected the activation of STAT3 signaling pathway. Additionally, we identified ZDHHC15 as an independent prognostic biomarker in glioma, and higher expression of ZDHHC15 predicted a poorer prognosis in glioma patients.

**Conclusion:**

Our findings suggest that ZDHHC15 promotes glioma malignancy and can serve as a novel prognostic biomarker for glioma patients. Targeting ZDHHC15 may be a promising therapeutic strategy for glioma.

**Supplementary Information:**

The online version contains supplementary material available at 10.1186/s12885-023-10883-6.

## Background

Glioma is a highly aggressive and deadly tumor in the adult central nervous system, accounting for approximately 80% of all malignant primary brain tumors [1–3]. According to the 2021 World Health Organization (WHO), glioma is classified into grades 2–4 based on its degree of malignancy [4]. Glioblastoma (GBM), the most common and lethal glioma, has a 5-year relative survival rate of less than 5% [5, 6]. The current treatment approach for glioma patients involves maximal safe border surgical resection and adjuvant chemoradiotherapy [7–9]. However, despite receiving these therapies, the median survival of GBM patients is only 14.6 months [10]. One of the significant characteristics of glioma cells is the ability to proliferate and migrate continuously, leading to incomplete tumor resection and postoperative tumor recurrence [11, 12]. This phenomenon is a major contributor to the poor prognosis of glioma patients [13]. Therefore, it is critical to investigate the underlying mechanisms of glioma progression and identify potential targets for future clinical treatment.

S-palmitoylation is a dynamic and reversible post-translational modification of proteins that owns significant implications for the accumulation, secretion, localization, stability, and function of proteins in cells by modulating their membrane affinity [14]. In mammals, protein palmitoylation is catalyzed by palmitoyltransferases (PATs), a family of enzymes known as ZDHHCs, which consists of 23 members (ZDHHC1-ZDHHC24, with the exception of ZDHHC10) that share a conserved Zinc finger DHHC-type domain [15]. PATs catalyze the transfer of palmitic acid (C16:0) or other long-chain saturated fatty acids to internal cysteine residues of proteins through unstable thioester linkages, leading to alterations in their physiological properties [16]. Emerging evidence suggests that PATs-mediated palmitoylation of proteins are implicated in carcinogenesis, tumor cell growth, survival, and treatment resistance. Recent studies have highlighted the widespread involvement of ZDHHCs in disease progression. For instance, in T_H_17 cells, the palmitoylation of STAT3 mediated by ZDHHC7 and the depalmitoylation of p-STAT3 by acylprotein thioesterase 2 (APT2) create a cycle that enhances the activation of STAT3 signaling pathway and promotes colitis [17]. Palmitoylation of the melanocortin-1 receptor (MC1R) by ZDHHC13 activates MC1R signaling, leading to increased pigmentation, controlling senescence, and preventing melanomagenesis. Upregulation of ZDHHC13 or inhibition of depalmitoylation of MC1R may be of potential in preventing melanoma progression [18]. In ovarian cancer, the membrane protein claudin-3 (CLDN3) promotes tumorigenesis and progression. ZDHHC12 catalyzes the palmitoylation of CLDN3, contributing to its stability and subcellular localization, thereby exerting its pro-tumor function [19].

In our previous study, we found that ZDHHC4, ZDHHC9, ZDHHC12, ZDHHC15, and ZDHHC23 were abnormally expressed in glioma compared to normal brain tissues. Specifically, we found that ZDHHC12 promotes glioma cell proliferation and the ZDHHCs-specific inhibitor, 2-bromopalmitate, significantly inhibits glioma cell proliferation [20]. It has been reported that ZDHHC15 is related to mental retardation associated with the X chromosome [21]. Previous research has also linked ZDHHC15 to the regulation of dendritic growth and formation and maturation of excitatory synapses [22, 23]. Nevertheless, the expression patterns and underlying biological functions of ZDHHC15 in glioma have not been fully explored. Therefore, the purpose of this study is to investigate whether ZDHHC15 promotes the progression of glioma or serves as a novel prognostic biomarker for glioma patients.

In this study, we first evaluated the mRNA and protein expression profile of ZDHHC15 in patients with glioma using both public datasets and clinical glioma samples. Subsequently, we explored the potential biological functions of ZDHHC15 in glioma through relevant enrichment analyses. To verify the functions and mechanisms of ZDHHC15 in the tumor progression of glioma cells, we performed ZDHHC15-siRNA knockdown and overexpression vector transfection experiments. Finally, we employed Kaplan-Meier and Cox proportional hazards regression analyses to evaluate the prognostic value of ZDHHC15 in glioma patients based on clinical information and ZDHHC15 expression.

## Materials and methods

### Data collection

To investigate the expression profile and potential biological functions of ZDHHC15 in glioma, the transcriptome data and clinical information of glioma patients were obtained from public databases, including the The Cancer Genome Atlas (TCGA) and Gravendeel databases (accessed via the GlioVis website http://gliovis.bioinfo.cnio.es), the Chinese Glioma Genome Atlas (CGGA) database (http://www.cgga.org.cn), and the GSE4290 database (accessed from the GEO database http://www.ncbi.nlm.nih.gov/geo/). In total, 1864 patients were included (687 from TCGA, 284 from Gravendeel including 8 normal brain tissues, 713 from CGGA including 20 normal brain tissues, and 180 from GSE4290 including 23 normal brain tissues). All bioinformatic datasets utilized in this study are publicly available.

### Functional analysis

Patients with glioma were classified into low- and high-expression subtypes based on the median expression of ZDHHC15. The “limma” R package was utilized to obtain the differentially expressed genes (DEGs) between the low- and high-expression groups, with thresholds of FDR < 0.05 and log2 (fold-change) > 1.0. To predict the potential functions of ZDHHC15, the Gene Ontology (GO) analysis, Kyoto Encyclopedia of Genes and Genomes [24] (KEGG) pathway analysis, and Gene Set Enrichment Analysis (GSEA) were performed using the “ClusterProfiler” R package. Additionally, the functional states of ZDHHC15 in glioma were explored using the CancerSEA website (http://biocc.hrbmu.edu.cn/CancerSEA/ home. Jsp).

### Survival prognostic analysis

The Kaplan-Meier curve was used for survival analysis, and the log-rank test was applied to determine the statistical differences. Univariate and multivariate Cox regression analyses were employed to identify the independent prognosis predictors. To obtain a nomogram, Cox proportional risk model was used in multivariable analyses, followed by the forward-LR method was used in univariate analysis with variables having a P < 0.05. The prognostic nomogram based on ZDHHC15 was constructed using the “RMS” R package. The calibration was graphically assessed with a calibration curve.

### Specimen collection

Tumor samples were collected from patients with glioma who underwent surgical treatment in the Neurosurgery Department of Zhongnan Hospital of Wuhan University between June 2016 and December 2021. The inclusion criteria were primary gliomas with no preoperative chemoradiotherapy. Immediately after removal, the tumor samples were frozen in liquid nitrogen and stored at -80℃ for further analysis. The grade of the tumor was determined according to the 2021 World Health Organization grading criteria for glioma by a pathologist [25]. A total of 186 glioma samples were collected, including grade 2, (n = 42), grade 3, (n = 33), and grade 4, (n = 111). Of these samples, 43 (collected from October 2020 to December 2021) were used for quantitative reverse transcription PCR assay to measure the mRNA level of ZDHHC15. Tissue microarrays were made from 143 tumor samples (collected from June 2016 to December 2019) to assess the expression of ZDHHC15 protein. Informed consent was obtained from each patient prior to surgery, and all experiments were approved by the Ethics Committee of Zhongnan Hospital.

### Cell culture

The human U87 and U251 glioma cell lines were obtained from the American Type Culture Collection (ATCC) and maintained in a humidified incubator at 37℃ with 5% CO_2_. The cells were cultured in Dulbecco’s Modified Eagle’s medium (DMEM; SH30022.01; Hyclone) supplemented with 10% fetal bovine serum (FBS, WISENT, Canada). The medium was refreshed twice a week.

### Cell transfection

Human ZDHHC15-specific siRNA was synthesized by Gene-Pharma (Shanghai, China). Using the jetPRIME® transfection reagent (Polyplus, France), a negative control (sense: 5’-UUCUCGAACGUGUCACGUTT-3’; antisense: 5’-ACUUGACACGUUCGGAGAATT-3’) or ZDHHC15-siRNA (sense: 5’-CCUUCCCUAUGAGGUCUAUTT-3’; antisense: 5’-AUAGACCUCAUAGGGAAGGTT-3’) was transfected into the U87 and U251 cells in 6-well plates. For ZDHHC15 overexpression, cDNA for ZDHHC15 was synthesized by Sangon Biotech (Shanghai, China) and inserted into the pcDNA3.1 vector. The ZDHHC15 overexpression plasmid and empty vector were transfected into glioma cells using Lipofectamine 3000 (Invitrogen, USA).

### CCK-8 assay

Cell viability was evaluated with a CCK-8 assay. After cells transfected for 48 h, U251 and U87 cells were cultured in 96-well plates with 2 × 10^3^ cells per well. At every time point, 10 µl cell counting kit-8 working solution (HUAYUCHENG, China) was added to each well. After one hour of incubation in a 37^o^C incubator, the absorbance of cells at 450 nm was measured. In addition, the standard curve of the CCK-8 experiment was plotted -- five quantitative gradients of 1000, 2000, 4000, 8000, 16,000 cells per well were done with three replicates per set. The OD450 values were detected 6 h after cell adherence. The results suggested that the OD450 value showed a linear relationship with the number of cells detected.

### EdU incorporation assay

Transfected cells were seeded into 24-well plates at 3 × 10^4^ cells per well. After 24 h, the cells were exposed to 10 µM EdU (5-ethynyl-2’-deoxyuridine; C0071S, BeyoClick™EdU-488, Beyotime) for 4 h, fixed, permeabilized, and stained with both the EdU and DAPI, according to the manufacturer’s protocol. Finally, cells were visualized under a fluorescent microscope.

### Transwell assay

After transfecting for 48 h, the cells were gently digested, and 0.2 ml serum-free medium containing 1.2 × 10^4^ U251 and U87 cells was added to the upper transwell chamber. The underside of the chamber was fraught with 0.6 ml DMEM containing 10% FBS. The cells were then incubated at 37 °C for 24 h, after which the chamber was removed and the cells that had not migrated in the chamber were wiped off. The chamber was fixed in 4% paraformaldehyde for 30 min and then stained in 0.1% crystal violet solution for another 30 min. Finally, the image of the cell migration through the chamber was captured using a microscope.

### Western blot assay

After washing three times with PBS buffer, the total proteins of U251 and U87 cells were extracted with RIPA lysate (Biosharp, China) on ice for 30 min. The protein concentration was measured using a BCA assay after centrifugation at 1.32 × 10^4^ rpm for 30 min at 4^0^ C. An equal amount of protein samples was separated by 10% SDS-PAGE and transferred to a PVDF membrane. After blocking with 5% skimmed milk for two hours, membranes were incubated with primary antibodies at 4^o^C for 18 h. The membranes were subsequently incubated with a goat anti-rabbit secondary antibody for 2 h. Finally, the bands were visualized using ECL solution (PUMOKE, China). The following antibodies were used: ZDHHC15 (1:1000, Sigma, HPA003618), Cyclin-B1 (1:1000, Cell Signaling Technology, #4138), Cyclin-D1 (1:1000, HUABIO, China, ET1601-31), MMP2 (1:2000, Abcam, ab92536), MMP9 (1:2000, Abcam, ab76003), GAPDH (1:5000, Servicebio, GB15004), STAT3 (1:1000, HUABIO, ET1607-38), p-STAT3 (1:1000, HUABIO, ET1603-40), the secondary antibody (1:10000, Biopmk, PMK-014-090 M). In this paper, all the blots were cut prior to hybridisation with antibodies during blotting.

### Quantitative real-time PCR

The total RNA of tumor samples and glioma cells was extracted using TRIzol reagent (Invitrogen, USA) following the manufacturer’s protocol. The RNA concentration was measured using a NanoDrop 2000 RNA (Thermo Fisher Scientific, USA). To obtain cDNA, PrimeScript RT Master Mix (Vazyme, China) was used to perform reverse transcription. For cDNA amplification, the following primers specific for ZDHHC15 were used: 5’-TGCCTGGTGACTGTTTTGAG-3’ (forward); 5’-ATGCAGGCCACAAAGAGAAG-3’ (reverse). GAPDH was used as an internal control, and the primers were: 5’-GGTGAAGGTCGGAGTCAACG-3’ (forward); 5’-CCATGTAGTTGAGGTCAATGAAG-3’ (reverse). An RT-qPCR was subsequently performed using SYBR Green Master Mix (R323-01, Vazyme, Nanjing, China) according to the manufacturer’s instructions. The relative expression of RNA was calculated using the 2^−∆∆Ct^ method.

### Immunohistochemistry

The paraffin-embedded sections of the glioma were first deparaffinized. Subsequently, the slices were subjected to boiling in the microwave oven using EDTA antigen repair solution (pH 8.0) for 3 min to repair the antigen and then cooled in pure water to normal temperature. The sections were then washed with PBST for 5 min three times. Blocking of the sections was carried out with peroxidase block solution for 15 min, followed by washing with distilled water. The slides were then covered with 5% goat serum for another 15 min. After blocking, the sections were washed with PBST for 5 min three times, and the ZDHHC15 primary antibody working solution (1:100) was added to the sections. The sections were then incubated in a humidified box at 4℃ overnight. The next day, the primary antibody on the sections was gently washed off with PBST, and the secondary antibody was incubated for 30 min. After washing the sections with PBS, DAB chromogenic agent was added. The sections were stained with hematoxylin and eosin dyes, air-dried, and coverslipped. In addition, the intensity of ZDHHC15 immunostaining in tumor cells was evaluated without knowledge of the clinical information of the sections. The proportion of stain was estimated based on a combined evaluation of the extent and intensity of staining. Scores ranging from 0 to 3 were assigned according to the percentage of positive tumor cells (0, ≤ 5%; 1, 6–25%; 2, 26–75%, and 3, > 75%). Staining intensity scores were categorized as negative (0 point), weak (1 point), intermediate (2 point), or high (3 point). The two scores were added up to give an overall score of 0–6: -, no expression (score 0–1); +, weak expression (score 2–3); ++, median expression (score 4); +++ indicates high expression (score 5–6).

### Statistical analysis

GraphPad Prism 8.0 software and R statistical software (Version 4.0.2) were used to perform statistical tests and plot graphs in this study. Quantitative data were expressed as mean ± standard deviation (SD). The unpaired t-test and Wilcoxon rank-sum test were applied to analyze the data. The relationship between clinicopathological information of glioma patients and the ZDHHC15 expression level was examined using the Pearson chi-square test. Grayscale measurement of protein bands and cell counting were performed with ImageJ Fiji software. The statistical significance was set at *P*<0.05.

## Results

### High expression of ZDHHC15 is related to the malignant phenotypes of glioma

**Fig. 1:**
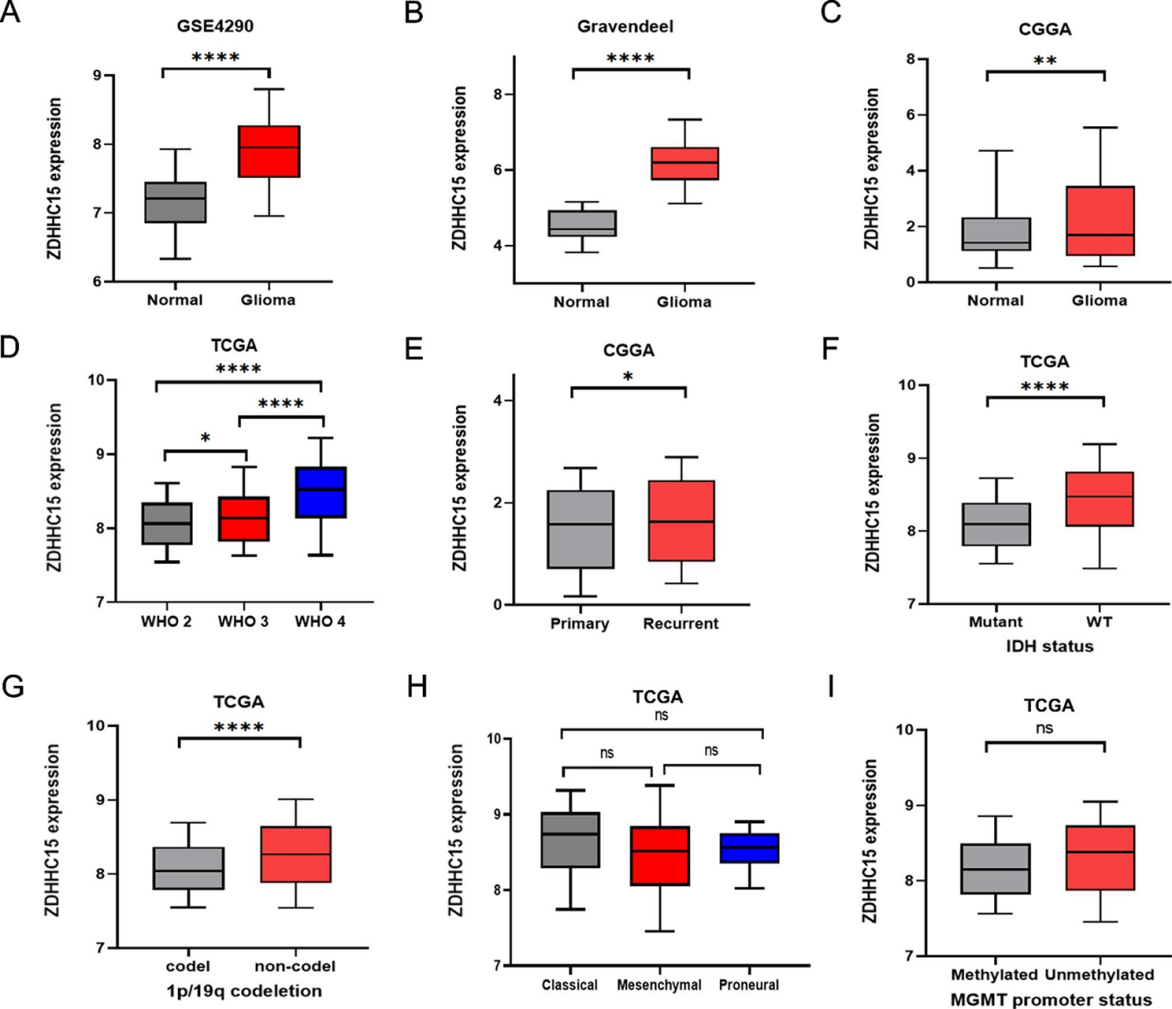
High ZDHHC15 mRNA is related to the malignant phenotypes of glioma. (**A-I**) Relative mRNA level of ZDHHC15 in normal brain tissues and glioma (**A-C**), grades 2–4 glioma (**D**), primary and recurrent glioma (**E**), IDH mutant and wild-type glioma (**F**), 1p/19q codel and non-codel glioma (**G**), three molecular subtypes of GBM (**H**), MGMTp methylated and unmethylated glioma (**I**). Student’s t-test, ^*^P < 0.05, ^**^P < 0.01, ^****^P < 0.0001

The study’s processes and details are presented in Figure S1. To investigate the expression profiles of ZDHHC15 in glioma, we first compared the transcriptional levels of ZDHHC15 between glioma tissues and normal brain tissues. Consistent with our previous finding, ZDHHC15 expression was significantly up-regulated in glioma (Fig. 1A-C). Subsequently, the association between ZDHHC15 expression level and glioma subtypes was explored. The results demonstrated that ZDHHC15 content was positively correlated with WHO grades in glioma (Fig. 1D). Furthermore, high ZDHHC15 expression was more prevalent in malignant glioma subtypes, including recurrence, IDH wild-type, and 1p/19q non-codeletion (Fig. 1E-G), which are considered markers of a poor prognosis [26, 27]. Conversely, there was no significant difference in ZDHHC15 mRNA expression among the three molecular subtypes of GBM and in the different methylation status of the MGMT promoter (Fig. 1H-I). These findings suggest that ZDHHC15 may be a valuable biomarker for malignant glioma.

**Table 1:** Association of ZDHHC15 protein expression with clinicopathological characteristics in human gliomas

ZDHHC15 intensity	Negative n (%)	Positive n (%)	P-value
Gender			0.9999
Male	55 (59.1)	29 (58.0)	
Female	38 (40.9)	21 (42.0)	
Age			0.0705
42<	29 (31.2)	8 (16.0)	
≥ 42	64 (68.8)	42 (84.0)	
Tumor location			0.4665
Frontal	29 (31.9)	19 (41.3)	
Temporal	15 (16.5)	5 (10.9)	
Others	47 (51.6)	22 (47.8)	
Tumor size, cm^3^			0.0262
<9.5	53 (57.0)	17 (37.0)	
≥ 9.5	40 (43.0)	29 (63.0)	
Symptoms at the diagnosis			0.0423
Epilepsy	22 (23.7)	4 (8.3)	
Headache	33 (35.5)	14 (29.2)	
Dizziness	15 (16.1)	11 (22.9)	
Limb weakness	7 (7.5)	10 (20.8)	
Others	16 (17.2)	9 (18.8)	
Karnofsky Performance Scale			0.1288
<90	36 (38.7)	25 (52.1)	
≥ 90	57 (61.3)	23 (47.9)	
Radio- and/or chemotherapy			0.7447
Yes	74 (90.2)	38 (88.4)	
No	8 (9.8)	5 (11.6)	
Tumor recurrence			0.1955
Yes	21(40.4)	13 (56.5)	
No	31 (59.6)	10 (43.5)	
IDH			0.0047
Mutate	40 (47.1)	11 (22.5)	
Wild-type	45 (52.9)	38 (77.5)	
Ki-67			0.0075
<10%	37 (43.5)	8 (17.0)	
10–20%	25 (29.4)	18 (38.3)	
>20%	23 (27.1)	21 (44.7)	
MGMTp methylation levels			0.330
Unmethylated	23 (40.4)	4 (26.7)	
Methylated	34 (59.6)	11 (73.3)	

### Correlation between ZDHHC15 expression and clinicopathologic characteristics in glioma

**Fig. 2:**
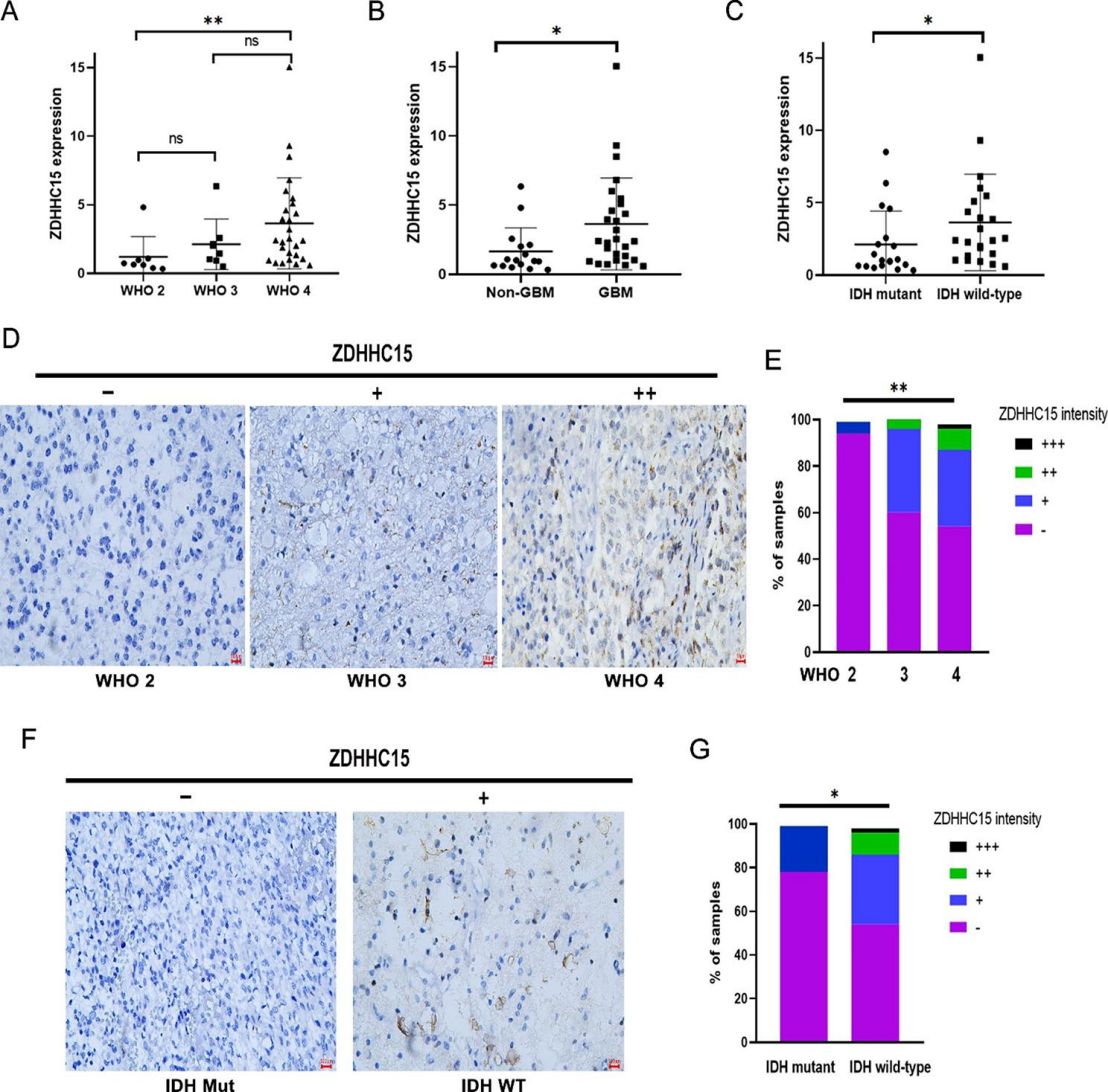
ZDHHC15 mRNA and protein levels in glioma tissues in our cohort. (**A-C**) Relative mRNA level of ZDHHC15 in grades 2–4 glioma (**A**), Non-GBM and GBM (**B**), IDH mutant and wild-type glioma (**C**), Student’s t-test. (**D-E**) Association of ZDHHC15 protein expression with tumor grades. (**F-G**) Association of ZDHHC15 protein expression with glioma IDH status. -, no expression (percentage of positive tumor cells ≤ 5% and staining intensity was negative); +, weak expression (percentage of positive tumor cells 6–25% and staining intensity was weak); ++, median expression (percentage of positive tumor cells 26–75% and staining intensity was intermediate); +++ indicates high expression (percentage of positive tumor cells >75% and staining intensity was high). Pearson chi-square test, ^*^P < 0.05, ^**^P < 0.01. Scale bars, 100 μm

To validate the results obtained from public datasets, 43 cases of human glioma tissues were collected to perform RT-qPCR assay (clinicopathologic information was presented in Table S1). Results confirmed that ZDHHC15 mRNA level was higher in malignant phenotypes of glioma, including grade 4 glioma, GBM, and IDH wild-type glioma (Fig. 2A-C). Additionally, we conducted an immunohistochemistry (IHC) experiment on a large number of glioma microarray samples (grades 2–4, n = 143; Table S2). The association between ZDHHC15 protein levels and clinicopathological information in glioma patients was presented in Table 1. Patients with high-grade glioma exhibited higher protein levels of ZDHHC15 compared to low-grade glioma (Fig. 2D-E). Furthermore, the proportion of ZDHHC15 protein positive expression in IDH wild-type glioma was higher than that in IDH mutant glioma (Fig. 2F-G). Further analyses of the glioma specimens revealed an important correlation between ZDHHC15 protein expression and Ki-67-positive cells (Table 1), suggesting that ZDHHC15 might be involved in glioma cell proliferation. In light of these results, we speculate that high expression of ZDHHC15 might play oncogenic roles in glioma.

### High ZDHHC15 expression is involved in cell cycle and migration of glioma

**Fig. 3:**
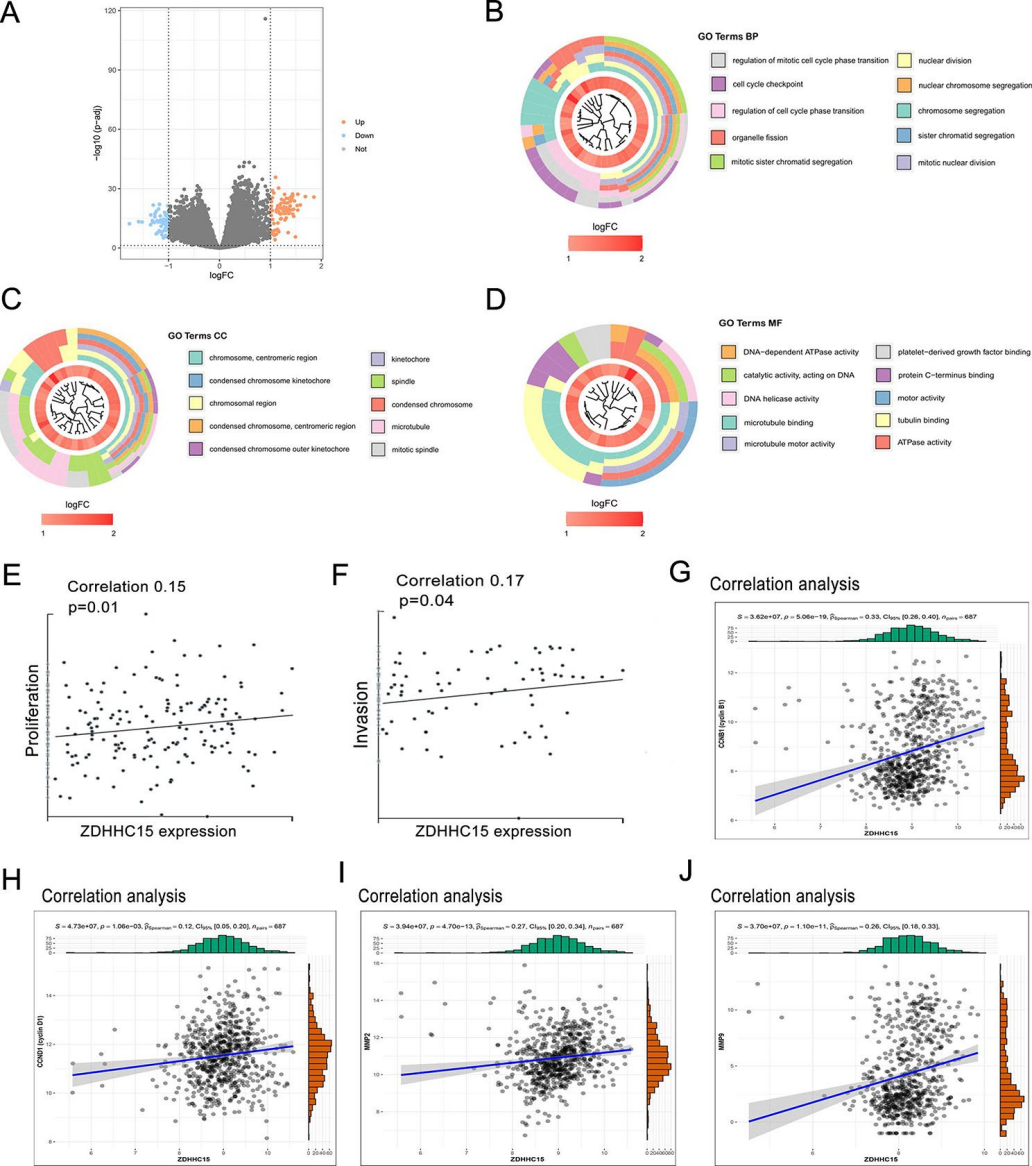
Potential biological functions of ZDHHC15 in glioma. (**A**) A volcano plot was generated to visualize the differentially expressed genes in the high and low ZDHHC15 expression groups in the TCGA database. (**B-D**) GO enrichment analysis of the up-regulated DEGs. (**E-F**) Analysis of the biological functions of ZDHHC15 in glioma in CancerSEA database. (**G-J**) Correlation analysis between ZDHHC15 and proliferation- or migration-related genes, including CCNB1/D1 and MMP2/9, were performed in the TCGA database. BP: biological process, CC: cellular component, MF: molecular function

To investigate the potential biological functions of ZDHHC15 in glioma, the data from TCGA and CGGA datasets were utilized to perform enrichment analysis. Glioma patients were divided into high- and low-expression groups based on the median expression level of ZDHHC15. We found that hundreds of genes were up-regulated in the ZDHHC15 high expression group compared to the low expression group (Fig. 3A; Figure S2A), which were used for GO and KEGG enrichment analysis. The results of GO and KEGG enrichment analysis revealed that biological processes related to proliferation and migration were enriched, such as nuclear division, cell cycle, regulation of cell cycle phase transition, and ECM-receptor interaction (Fig. 3B-D; Figure S2B-E). We further analyzed the biological function of ZDHHC15 using CancerSEA, a multifunctional website that explores different functional states of cancer cells at the single-cell level [28], and the results indicated that ZDHHC15 was positively correlated with tumor cell proliferation and invasion (Fig. 3E- F). Moreover, gene correlation analysis in TCGA and CGGA databases also verified that *ZDHHC15* was significantly associated with glioma cell proliferation- and migration-related genes (Fig. 3G-J; and Figure S2F-J). Taken together, these results suggest that high ZDHHC15 expression may be involved in glioma cell proliferation and migration.

### ZDHHC15 promotes the proliferation and migration of human glioma cells

**Fig. 4:**
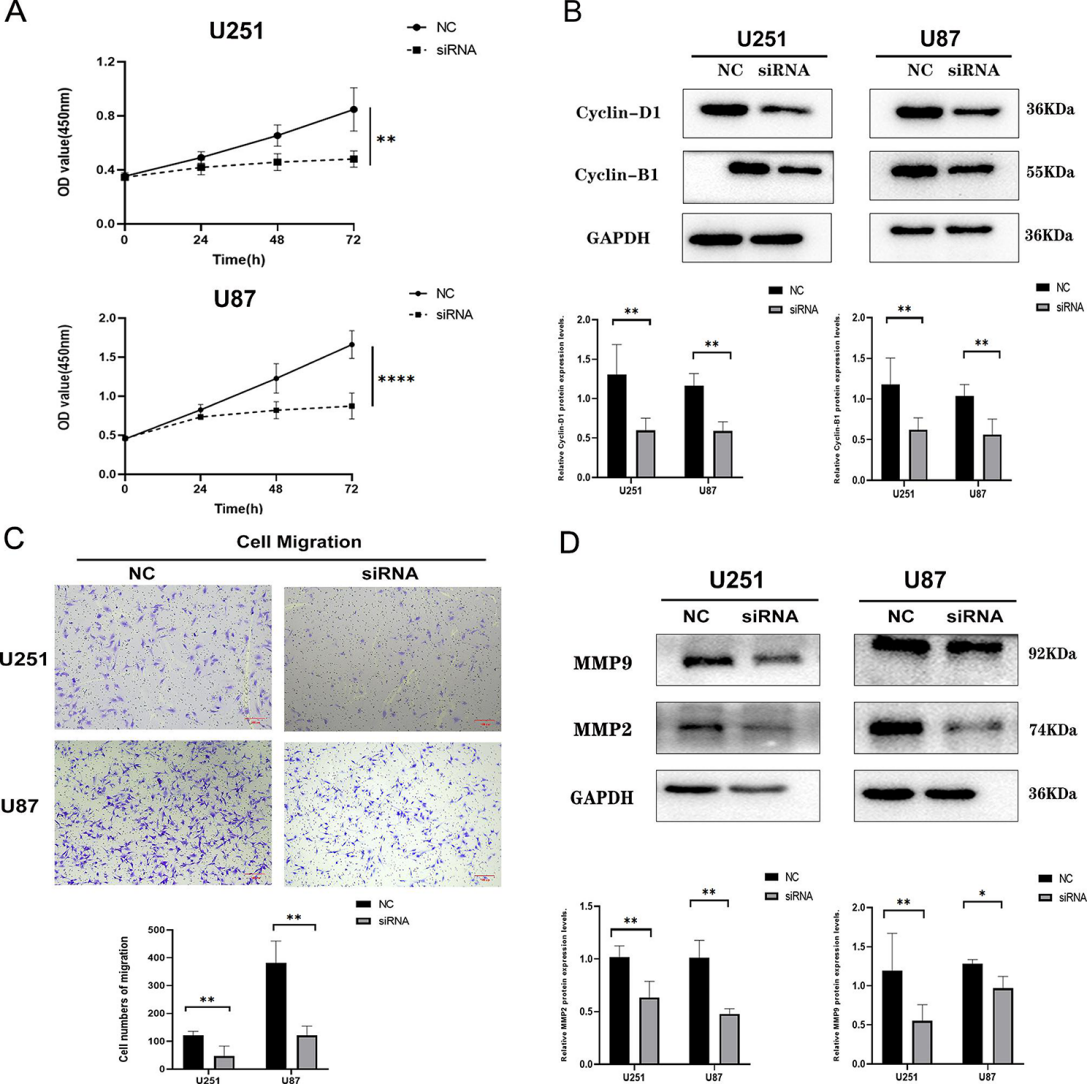
Knockdown of ZDHHC15 inhibits glioma cell proliferation. (**A**) A CCK-8 assay was used to evaluate the viability of glioma cells after the downregulation of ZDHHC15. (**B**) Cell cycle-related key proteins of U251 and U87 cells were detected by Western blotting assay after transfection of ZDHHC15 siRNA. (**C**) The effect of ZDHHC15 knockdown on cell migration of U251 and U87 cells was examined by Transwell assay. (**D**) Cell migration-related key proteins of U251 and U87 cells were detected by Western blotting assay after transfection of the ZDHHC15 siRNA. Student’s t-test. ^*^P < 0.05, ^**^P < 0.01, ^****^P < 0.0001. Scale bars, 100 μm

To validate the role of ZDHHC15 in glioma, ZDHHC15 siRNA was transfected into U251 and U87 cells and related experiments were performed. We designed three ZDHHC15-specific siRNAs and results showed that two of these siRNAs had an ideal silencing effect (Figure S3A) and produced the same functional effect (Figure S3B). Therefore, we selected one siRNA for further experiments(Figure S3C). Consistent with our bioinformatics analysis, the viability and cell numbers of U251 and U87 cells decreased significantly over 24, 48, and 72 h of continuous exposure following ZDHHC15-siRNA treatment (Fig. 4A, and Figure S4). Furthermore, the EdU assay results indicated a considerable restriction in the proliferation ability of glioma cells after ZDHHC15 knockdown (Figure S5). A reduction in the expression of cyclin-B1 and cyclin-D1 proteins was also observed in the ZDHHC15 siRNA groups (Fig. 4B). Moreover, we measured the effects of ZDHHC15 on glioma cell migration in U251 and U87 cells. Transwell assay results revealed that glioma cell migration rates were reduced following ZDHHC15 knockdown (Fig. 4C). Additionally, the expression of cell migration-related proteins, MMP2 and MMP9, was markedly decreased after the knockdown of ZDHHC15 (Fig. 4D).

**Fig. 5:**
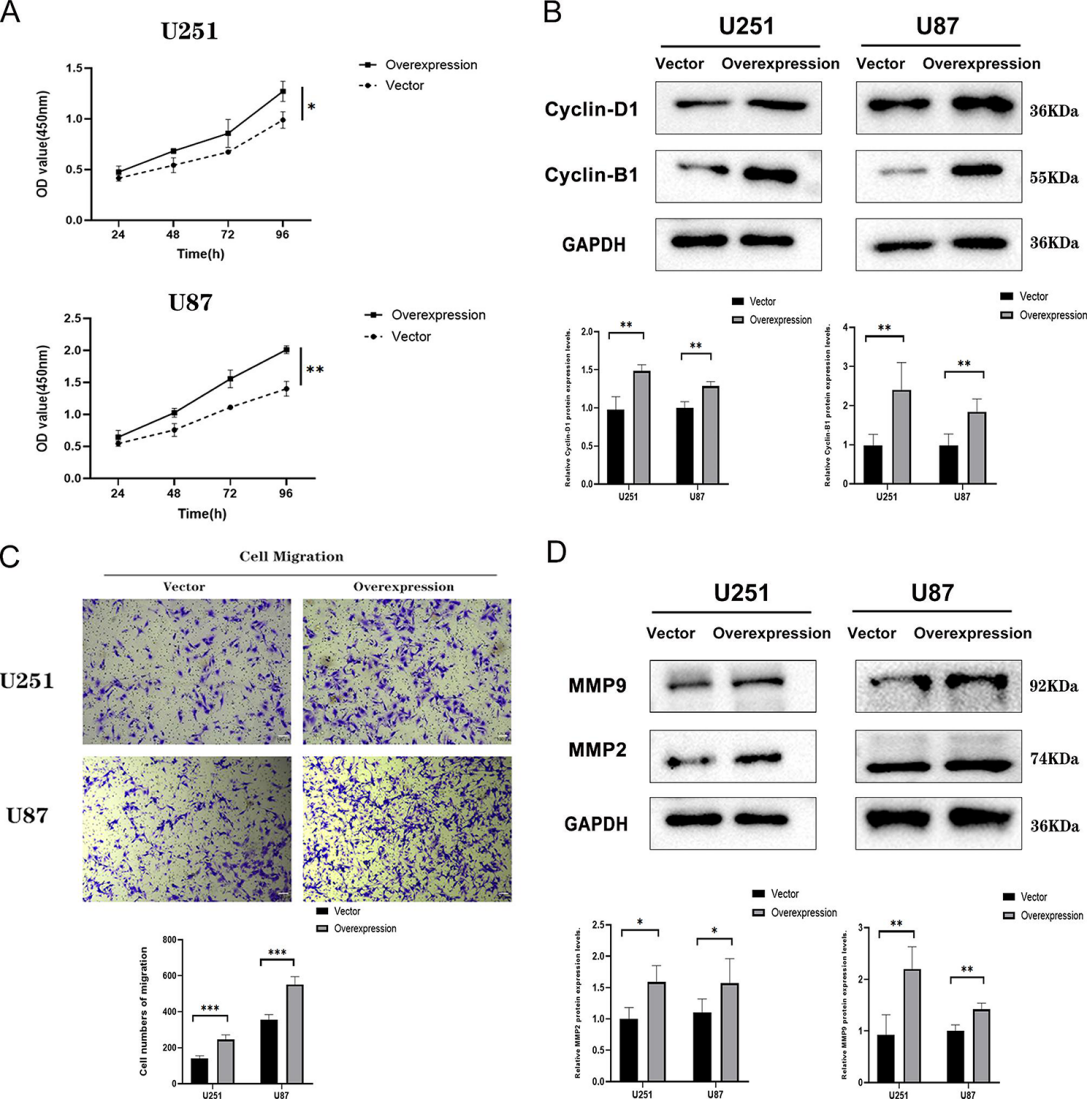
Overexpression of ZDHHC15 promotes glioma cell proliferation and migration. (**A**) The effect of ZDHHC15 overexpression on cell proliferation of U251 and U87 cells was examined by CCK-8 assay. (**B**) Cell cycle-related key proteins of U251 and U87 cells were detected by Western blotting assay after transfection of ZDHHC15 overexpression plasmid. (**C**) The effect of ZDHHC15 overexpression on cell migration of U251 and U87 cells was examined by Transwell assay. (**D**) Cell migration-related key proteins of U251 and U87 cells were detected by Western blotting assay after transfection of the ZDHHC15 overexpression plasmid. Student’s t-test. ^*^P < 0.05, ^**^P < 0.01, ^***^P < 0.001. Scale bars, 100 μm

To further verify the biological functions of ZDHHC15, we established glioma cell lines that overexpressed ZDHHC15 (Figure S3D). Consistent with above findings, glioma cells transfected with ZDHHC15 proliferated faster than cells transfected with the control vector (Fig. 5A, and Figure S4). The expression of cyclin-B1 and cyclin-D1 were up-regulated in ZDHHC15-transfected cells (Fig. 5B). To assess the effects of ZDHHC15 overexpression on cell migration in vitro, we conducted a cell migration assay. As expected, ZDHHC15-overexpression cells exhibited significantly increased migration rates and the migration-related proteins MMP2 and MMP9 were up-regulated compared to empty vector groups (Fig. 5C-D). Taken together, these results indicate that ZDHHC15 promotes the proliferation and migration of human glioma cells.

### ZDHHC15 activates the STAT3 signaling pathway

**Fig. 6:**
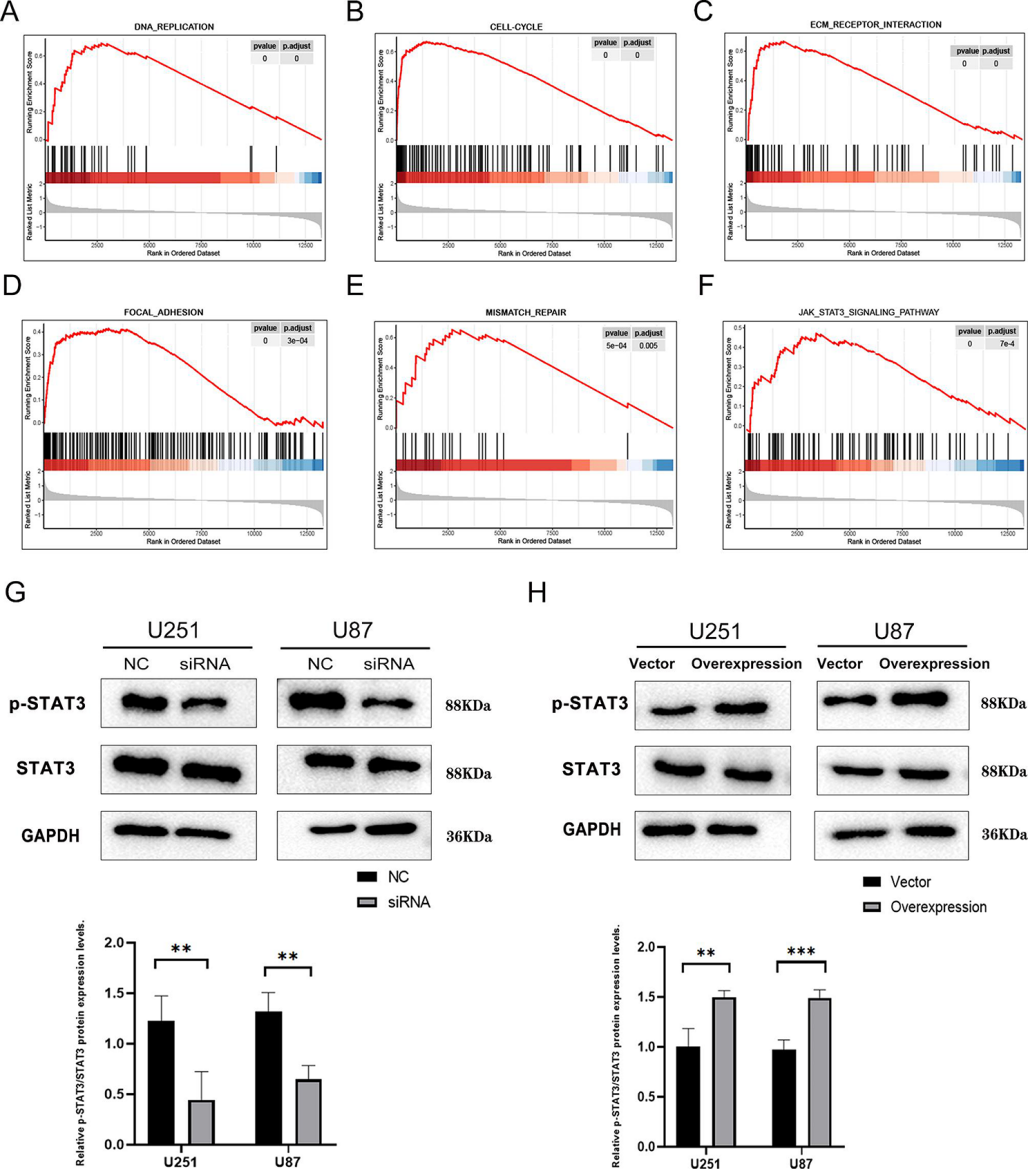
ZDHHC15 activates the STAT3 signaling pathway in glioma cells. (**A-F**) GSEA analysis of ZDHHC15 in glioma in the TCGA database. (**G-H**) STAT3 and p-STAT3 proteins of U251 and U87 cells were detected by Western blotting assay after transfection of ZDHHC15-siRNA or ZDHHC15 overexpression plasmid. Student’s t-test. ^**^P < 0.01, ^***^P < 0.001

After validating the biological functions of ZDHHC15 in glioma, the underlying mechanism of ZDHHC15 in glioma was explored. Our gene set enrichment analysis (GSEA) results revealed a significant enrichment of the STAT3 signaling pathway in high ZDHHC15 expression in glioma (Fig. 6A-F, and Figure S6). Signal Transducer and Activator of Transcription 3 (STAT3) is an important transcription factor that regulates various cellular processes, including cell proliferation, migration, apoptosis, angiogenesis, and immune response. Activation of the STAT3 signaling pathway has been implicated in the development of numerous human cancers [29]. Results from the western blotting showed that knockdown of ZDHHC15 significantly decreased the levels of phosphorylated STAT3 (p-STAT3) in U251 and U87 cells, while total STAT3 protein levels remained unchanged (Fig. 6G). Conversely, overexpression of ZDHHC15 in glioma cell lines resulted in increased p-STAT3 levels (Fig. 6H). In addition, we used a common STAT3 inhibitor in the ZDHHC15 overexpression assay, Stattic, which selectively inhibited activation, dimerization, and nuclear translocation of STAT3 by binding to its Src homology 2 (SH2) domain [30]. As expected, the proliferation and migration of glioma cells overexpressed with ZDHHC15 were significantly inhibited after Stattic administration (Figure S7). Taken together, these findings provide compelling evidence that ZDHHC15 could activate the STAT3 signaling pathway.

### High expression of ZDHHC15 is an independent and unfavorable prognostic biomarker in glioma patients

**Fig. 7:**
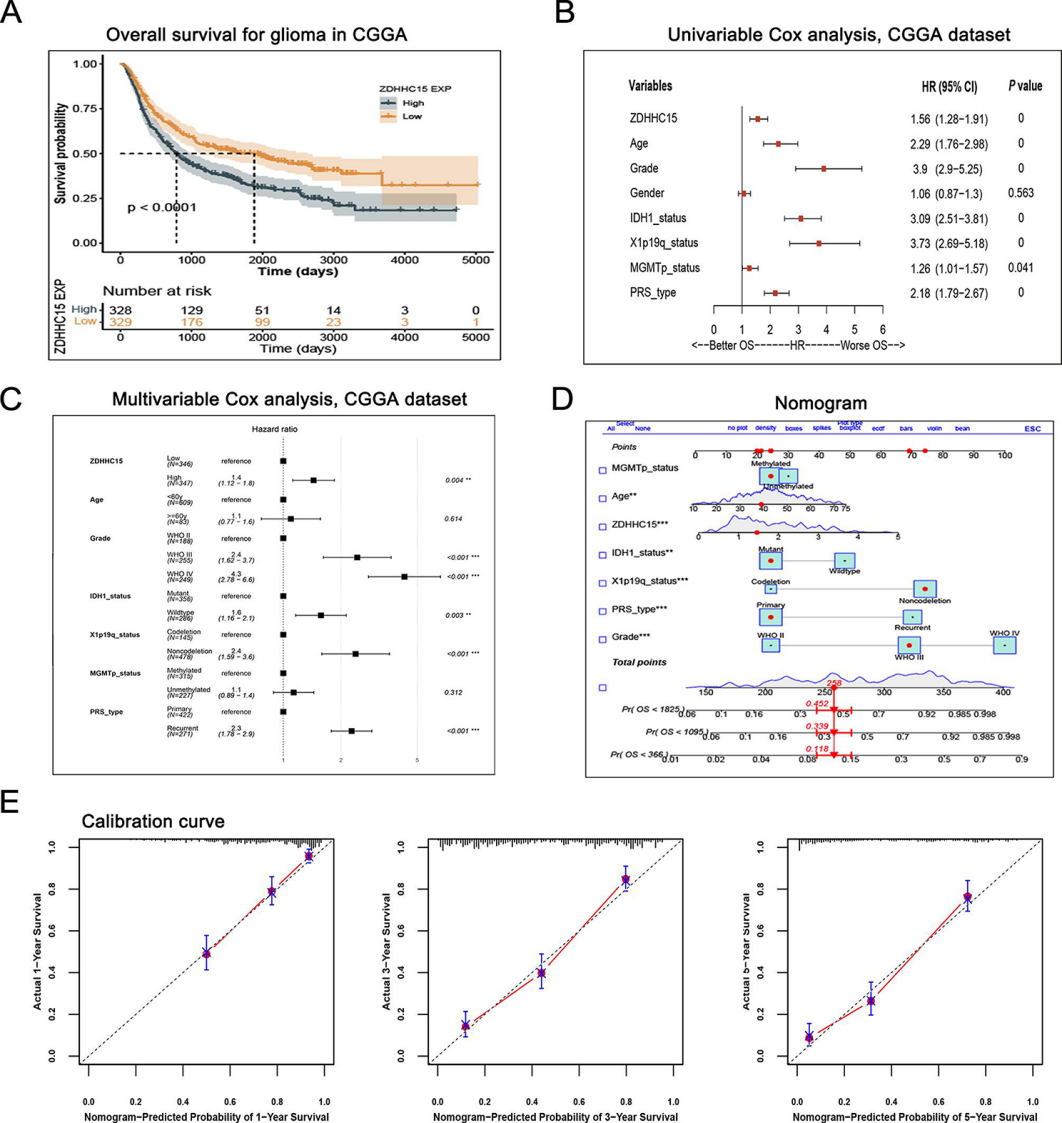
Prognostic value of ZDHHC15 in patients with glioma. (**A**) High expression of ZDHHC15 predicted poor outcomes in patients with glioma in the CGGA database. (**B**) Univariable Cox analyses of ZDHHC15 among glioma patients. (**C**) Multivariable Cox analyses of ZDHHC15 among glioma patients. (**D**) Nomogram based on risk scores and other clinical indicators in the CGGA database. (**E**) Calibration curves were used to predict the 1,3,5 years survival rate of glioma patients in the CGGA database

Our data demonstrated that glioma patients with ZDHHC15 protein-positive expression had worse progression-free survival and overall survival (Figure S8A-B). To further confirm the prognostic value of ZDHHC15 in glioma patients, we compared the prognosis of glioma patients with high and low-ZDHHC15 expression in the CGGA database. The results showed that glioma patients with higher ZDHHC15 expression exhibited poorer prognosis compared to that with lower ZDHHC15 expression (Fig. 7A). Furthermore, high ZDHHC15 expression was significantly associated with worse prognosis in patients with IDH1 wild-type and mutant glioma, 1p/19q non-codeletion glioma, MGMT promoter methylated and non-methylated glioma, and primary and recurrent glioma (Figure S8C-F).

In addition, univariate and multivariate Cox analysis models were applied to assess the prognostic ability of ZDHHC15. Results showed that high ZDHHC15 expression was a high-risk indicator and an independent prognostic marker in patients with glioma (Fig. 7B-C). Finally, we combined ZDHHC15 with common clinical risk factors to create a nomogram, which provides a model to quantitatively predict the prognosis of glioma patients (Fig. 7D). The 1, 3, and 5-year expected survival rates of glioma patients were calculated, and the calibration curve almost coincided with the ideal curve (Fig. 7E). Collectively, these results reveal that ZDHHC15 is a prognostic biomarker in glioma patients and our nomogram can well predict the prognosis of glioma patients.

## Discussion

Glioma is the most common primary malignant tumor in adults and is associated with high mortality rates. Although it accounts for less than 1% of newly diagnosed tumors, its significant morbidity and mortality impose a significant health burden [26]. Studies suggest that ZDHHC family enzymes are widely involved in glioma development and progression. For instance, recent study demonstrated that ZDHHC9-mediated palmitoylation of Glucose transporter 1 (GLUT1) plays a decisive role in the localization of GLUT1, and palmitoylated GLUT1 can promote glioblastoma glycolysis and tumorigenesis [31]. ZDHHC5-mediated palmitoylation of EZH2 induces the occurrence and progression of p53 mutant glioma [32]. Furthermore, in mesenchymal glioblastoma, ZDHHC18 enhances the stability of BMI1 to promote the survival of glioblastoma stem cells (GSCs) in stressful microenvironments [33]. However, the function of ZDHHC15 in glioma progression is not well understood. In this study, we found that ZDHHC15 expression is significantly associated with glioma malignant phenotypes. Knockdown or overexpression of ZDHHC15 in glioma could regulate tumor cell proliferation and migration by targeting the STAT3 signaling pathway. High expression of ZDHHC15 is associated with poor prognosis and ZDHHC15 is an independent prognostic biomarker in patients with glioma.

ZDHHC15 is a protein palmitoyltransferase encoded by the zine finger DHHC domain-containing protein 15 gene located at Xq13.3 in humans. ZDHHC15-deficient mice exhibit increased novelty-induced locomotion, extracellular dopamine in the striatum, and sensitivity to amphetamine, all of which are traits associated with some psychiatric illnesses [34]. Additionally, ZDHHC15 gene mutation has been linked to hypotonic cerebral palsy, autism, epilepsy, and intellectual disabilit [35], suggesting that ZDHHC15 plays an essential role in the central nervous system and is associated with psychiatric disorders. However, the relationship between ZDHHC15 and tumors had hardly been studied. In this study, we found that ZDHHC15 mRNA levels were up-regulated in glioma tissues compared to normal brain tissues based on data from public databases and our clinical glioma samples. High expression of ZDHHC15 was positively associated with malignant phenotypes of glioma, particularly in WHO high-grade, IDH wild-type, 1p/19q non-codeletion, and recurrence subtypes. Compared with clinical characteristics classification (age, gender, and others), these subtypes could be better used to assess and manage glioma patients [26, 36]. We further verified these findings through the IHC assay, which showed that high ZDHHC15 protein expression was significantly correlated with WHO high-grade glioma and IDH wild-type glioma. Our data also suggested that ZDHHC15 was linked to the progression of glioma and might have potential clinical value in the management of glioma patients as there was a protein expression correlation between ZDHHC15 and Ki-67, which is used to determine the proliferative activity of glioma cells and assess grade and malignancy [37]. Besides glioma, we found that ZDHHC15 was aberrantly expressed in other tumors (Figure S9A). However, unlike glioma, ZDHHC15 expression was downregulated in ovarian serous cystadenocarcinoma (OV), cervical squamous cell carcinoma and endocervical adenocarcinoma (CESC), and uterine corpus endometrial carcinoma (UCEC). Interestingly, ZDHHC15 expression increased consistently with higher grades in UCEC, and the prognosis of UCEC patients with high ZDHHC15 expression was poorer than the low group (Figure S9B-C). These data suggested that ZDHHC15 might play a pro-tumor function in UCEC, which warrants further research in the future.

Infiltrative growth and continuous proliferation of glioma cells render the disease incurable, making it imperative to explore the underlying mechanisms of malignant progression. Our results revealed that glioma cell proliferation and migration-related processes were highly enriched in the high ZDHHC15 expression group. Single-cell functional analysis of tumors showed an association between ZDHHC15 and tumor cell proliferation and invasion. In vitro experiments confirmed that knockdown of ZDHHC15 suppressed glioma cell proliferation and migration, while overexpression of ZDHHC15 promoted proliferation and migration.

Furthermore, we also investigated the molecular mechanism of ZDHHC15 in glioma progression. In glioblastoma stem cells (GSCs), it had been reported that local anesthetics suppressed the STAT3 signaling pathway by inhibiting ZDHHC15 [38]. Our GSEA analysis results showed that ZDHHC15 was enriched in the JAK/STAT3 signaling pathway. STAT3 had been found to be over-activated and played a tumor-promoting role in a variety of human cancers because STAT3 could act as a transcription factor to promote the expression of cyclin-B1, cyclin-D1, MMP2, MMP9 and other pro-tumor proteins [29]. Moreover, STAT3 was hyperactivated in glioma and excessive activation of STAT3 promoted gliomagenesis, cell survival, proliferation, migration, angiogenesis, and other biological effects [39]. Knockdown and overexpression of ZDHHC15 inhibited and activated the STAT3 signaling pathway, respectively. These findings suggest that ZDHHC15 promotes the malignant progression of glioma by activating the STAT3 signaling pathway. However, the specific molecular mechanism of ZDHHC15 activation of the STAT3 signaling pathway is still unknown. Recent research has shown that STAT3 is palmitoylated by ZDHHC7, which facilitates STAT3 signaling pathway activation by translocating it from the cytoplasm to the cell membrane [17]. However, other ZDHHCs, such as ZDHHC17, have been found to accelerate glioma progression by interacting with MAP2K4, and this function may depend on the ZDHHC17 ankyrin-repeat domain, rather than its palmitoyltransferase activity [40]. Therefore, further study is needed to elucidate the specific molecular mechanism of ZDHHC15 activation of STAT3 signaling pathway.

Based on our bioinformatics analysis and experimental results, we hypothesized that ZDHHC15 is a prognostic marker in glioma patients. As expected, we observed poor overall survival in glioma patients with high ZDHHC15 expression compared to the low ZDHHC15 expression groups. Univariate and multivariate Cox analysis further confirmed that ZDHHC15 is an independent prognostic biomarker. Notably, emerging evidence has shown that ZDHHCs are associated with the prognosis of tumor patients, but the same ZDHHCs might exhibit opposite prognostic roles in different tumors. For example, overexpression of ZDHHC11 is strongly linked to high grade, disease progression, and unfavorable prognosis in bladder cancer [41], while patients with high ZDHHC11 expression exhibit favorable prognostic features in glioma [20]. In kidney renal clear cell carcinoma, ZDHHC15 is involved in immune regulation and negatively regulates the JAK/STAT signaling pathway, and patients with low ZDHHC15 expression present dismal overall survival [42]. However, in our study, glioma patients with high ZDHHC15 expression showed poor overall survival. These results suggest that the prognostic functions of ZDHHCs might vary by tumor type. Moreover, we constructed a nomogram to quantitatively assess prognostic risk in patients with glioma, and the 1,3, and 5-year survival lines almost overlapped with the calibration curves, indicating that our nomogram can well predict the prognosis of patients with glioma.

## Conclusion

In conclusion, this study provides the first comprehensive analysis of the expression pattern of ZDHHC15 in glioma and establishes a clear association between high ZDHHC15 expression and malignant glioma phenotype. Our results demonstrate that ZDHHC15 plays a critical role in promoting the proliferation and migration of glioma cells via activation of the STAT3 signaling pathway. Importantly, our findings suggest that ZDHHC15 may serve as a new prognostic biomarker for glioma patients, and targeting ZDHHC15 may offer a promising strategy for the treatment of glioma.

## Electronic supplementary material

Below is the link to the electronic supplementary material.

Supplementary Material 1

## Data Availability

The raw data for this study will be provided by the authors upon reasonable request. Requests for access to the dataset should be sent to lizhiqiang@whu.edu.cn.

## Abbreviations

CCK-8: Cell count kit-8
CESC: Cervical squamous cell carcinoma and endocervical adenocarcinoma
CGGA: Chinese Glioma Genome Atlas
GBM: Glioblastoma
GEO: Gene Expression Omnibus
IHC: Immunohistochemistry
OV: Ovarian serous cystadenocarcinoma
TCGA: The Cancer Genome Atlas
UCEC: Uterine Corpus Endometrial Carcinoma
