# Supplementary material for: ZDHHC15 promotes glioma malignancy and acts as a novel prognostic biomarker for patients with glioma
Source: BMC Cancer. 2023 May 9;23:420. doi: 10.1186/s12885-023-10883-6 (PMC10169355; doi:10.1186/s12885-023-10883-6)

ZDHHC15 promotes glioma malignancy and acts as a novel prognostic biomarker for patients with glioma

Zhen-Yuan Liu^1†^, Tian Lan^1†^, Feng Tang^1†^, Yong-Ze He^1^, Jin-Sheng Liu^1^, Jin-Zhou Yang^1^, Xi Chen^1^, Ze-Fen Wang^2*^, Zhi-Qiang Li^1*^

1 Brain Glioma Center & Department of Neurosurgery, Zhongnan Hospital of Wuhan University, Wuhan, China

2 Department of Physiology, Wuhan University School of Basic Medical Sciences, Wuhan, China

† These authors have contributed equally to this work

* Corresponding authors: Ze-Fen Wang [wangzf@whu.edu.cn；Zhi-Qiang Li lizhiqiang@whu.edu.cn](mailto:wangzf@whu.edu.cn；lizhiqiang@whu.edu.cn)

**
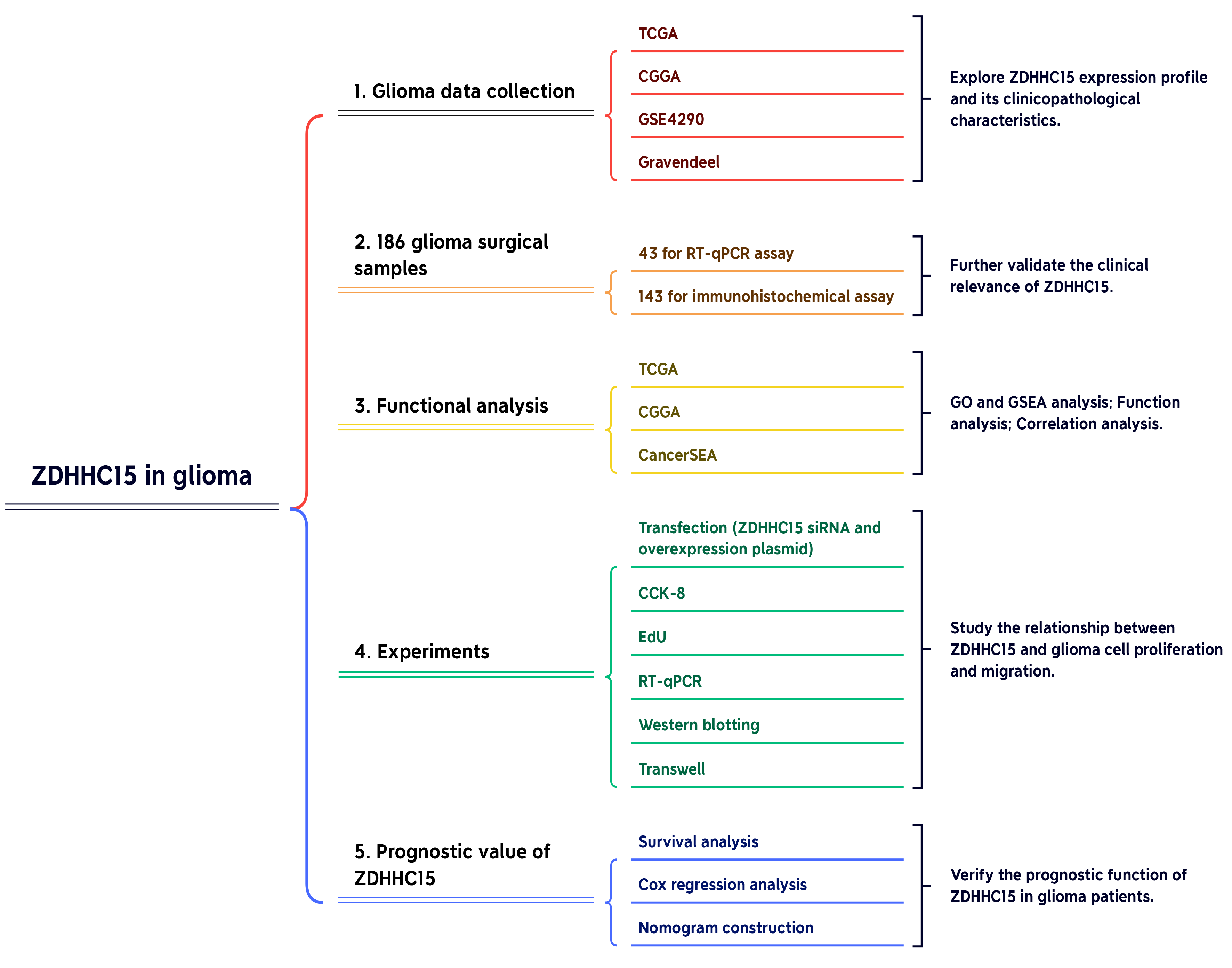
**

Figure S1. A diagram for the details of the present study.

**
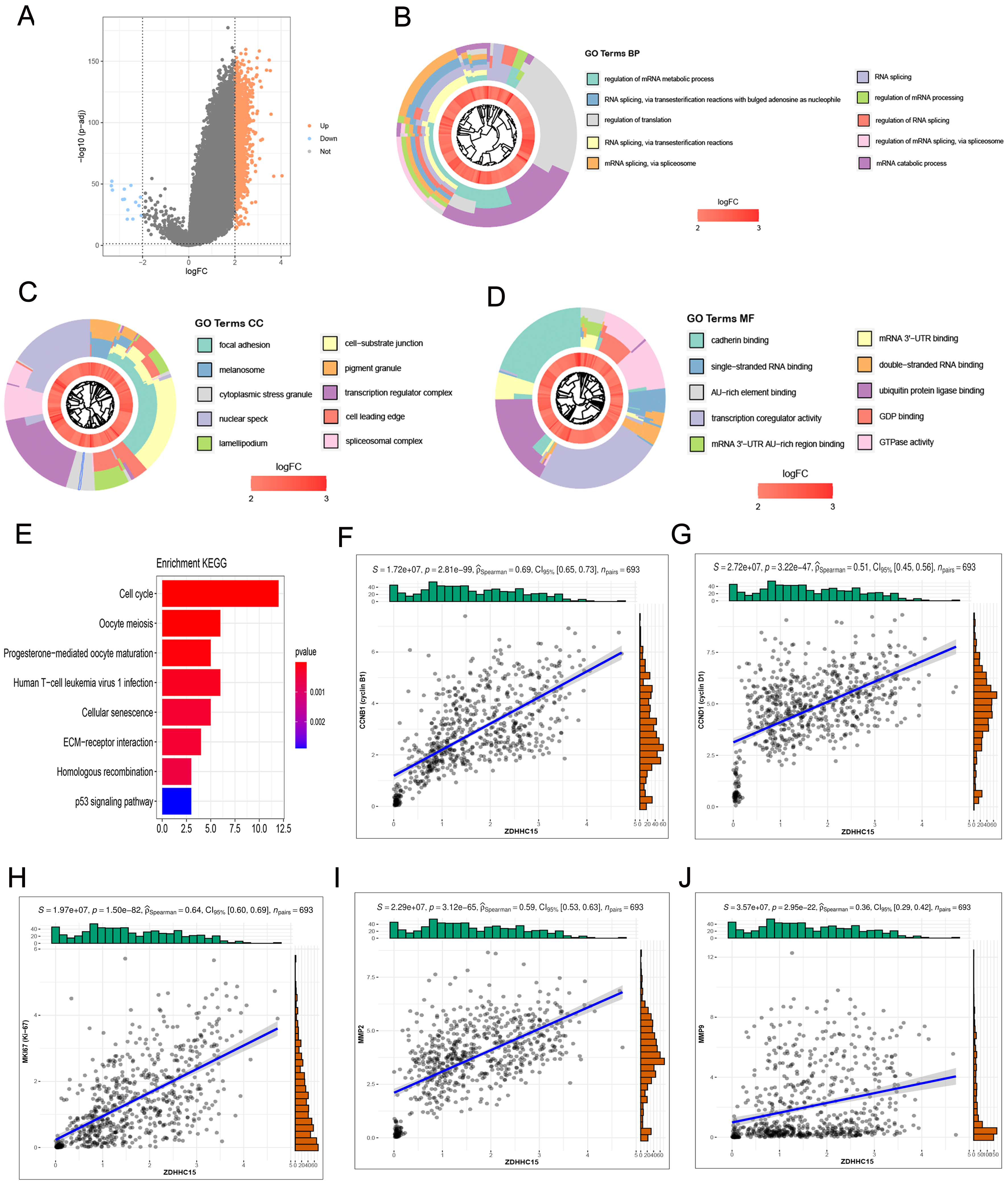
**Figure S2. Potential biological functions of ZDHHC15 in glioma. (A) A volcano plot was generated to visualize the differentially expressed genes in the ZDHHC15-high and low expression groups in the CGGA database. (B-E) GO and KEGG enrichment analysis of the up-regulated DEGs. KEGG analysis received formal permission from Kanehisa laboratories. (F-J) Correlation analysis between ZDHHC15 and proliferation- or migration-related genes, including CCNB1/D1, MKi-67, and MMP2/9, were performed in the CGGA database. BP: biological process, CC: cellular component, MF: molecular function.


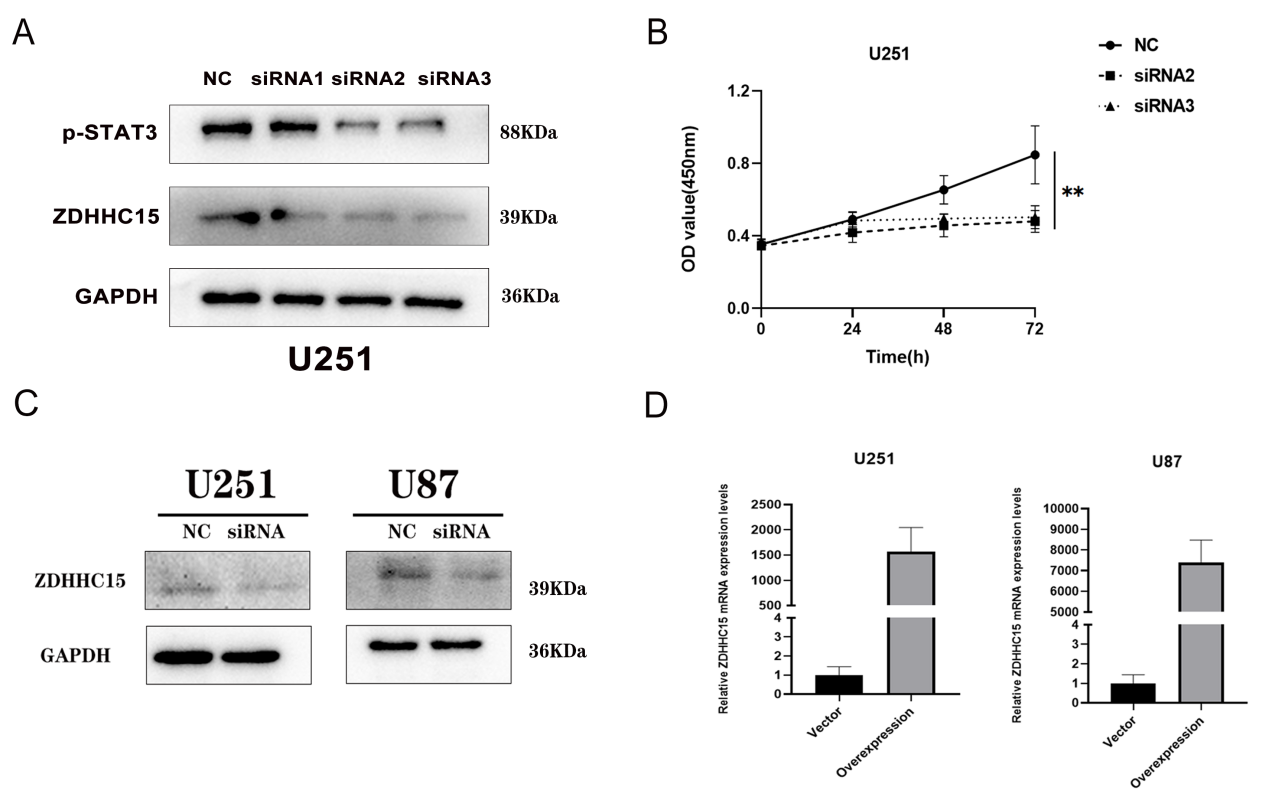
Figure S3. (A) Examination of the effectiveness of three specific siRNAs for silencing ZDHHC15. (B) Efficacy of ZDHHC15-siRNA2 and siRNA3 in inhibiting the proliferation of glioma cells. (C, D) Models of ZDHHC15 knockdown and overexpression in U251 and U87 cells. Student’s t-test, ^**^*P* ＜ 0.01.

**
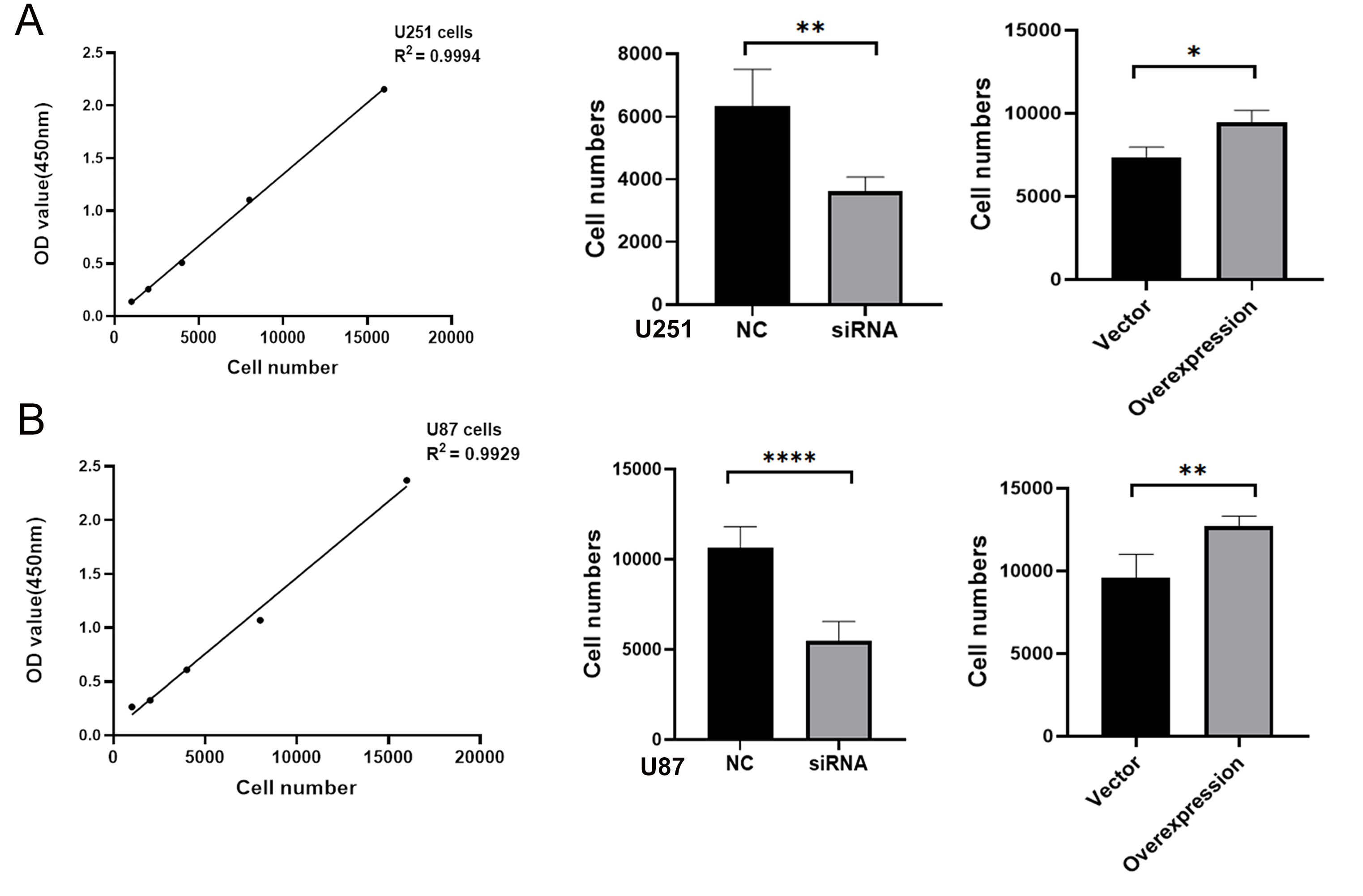
**Figure S4. Standard curve and cell numbers of CCK-8 experiment for U251(A) and U87 (B) cells. Student’s t-test, ^*^*P* ＜ 0.05, ^**^*P* ＜ 0.01, ^****^*P* ＜ 0.0001.

**
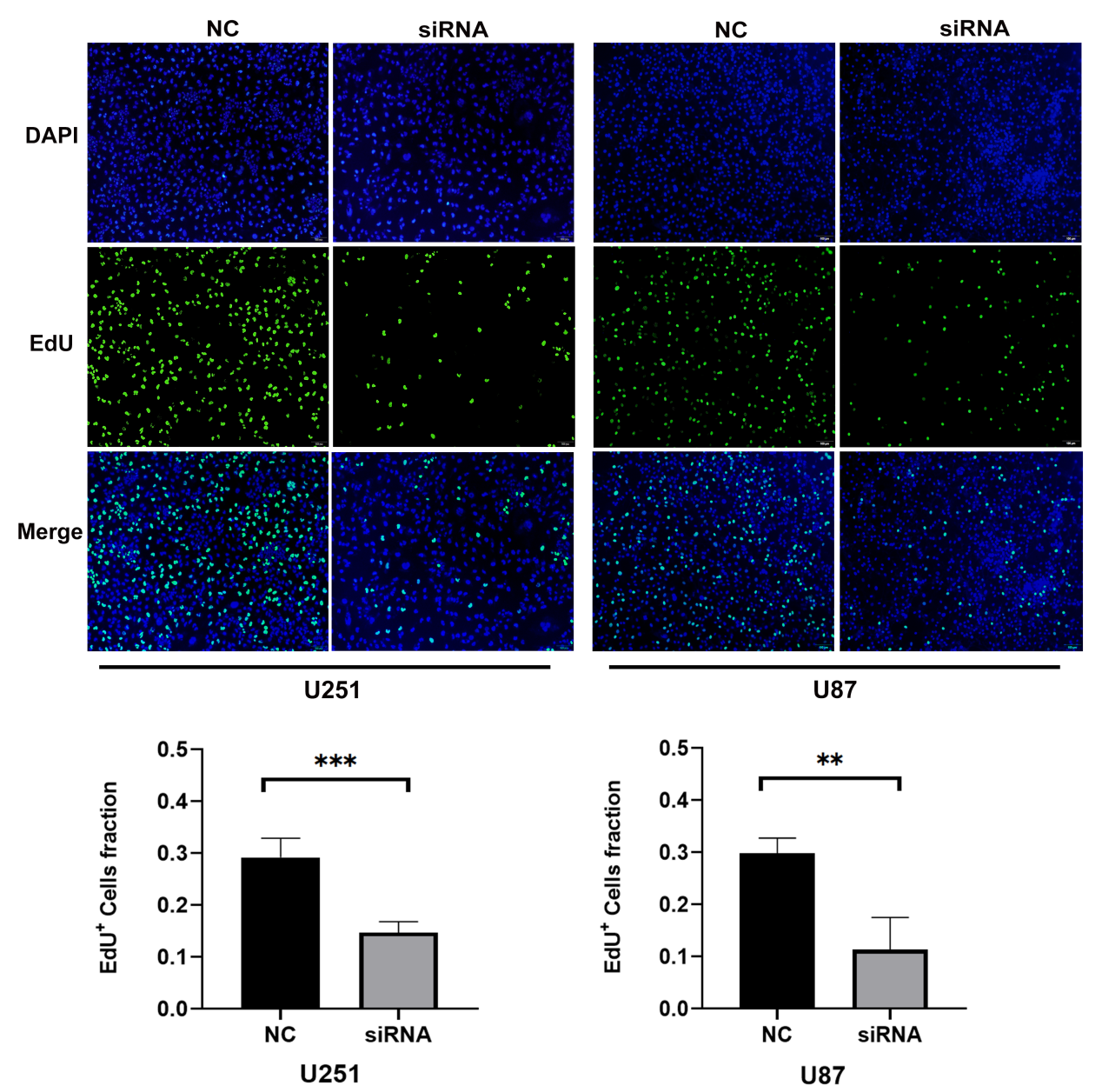
**

Figure S5. An EdU assay was used to evaluate the proliferation of glioma cells after the downregulation of ZDHHC15. Scale bars, 100μm, Student’s t-test, ^**^*P* ＜ 0.01, ^***^*P* ＜ 0.001.

.


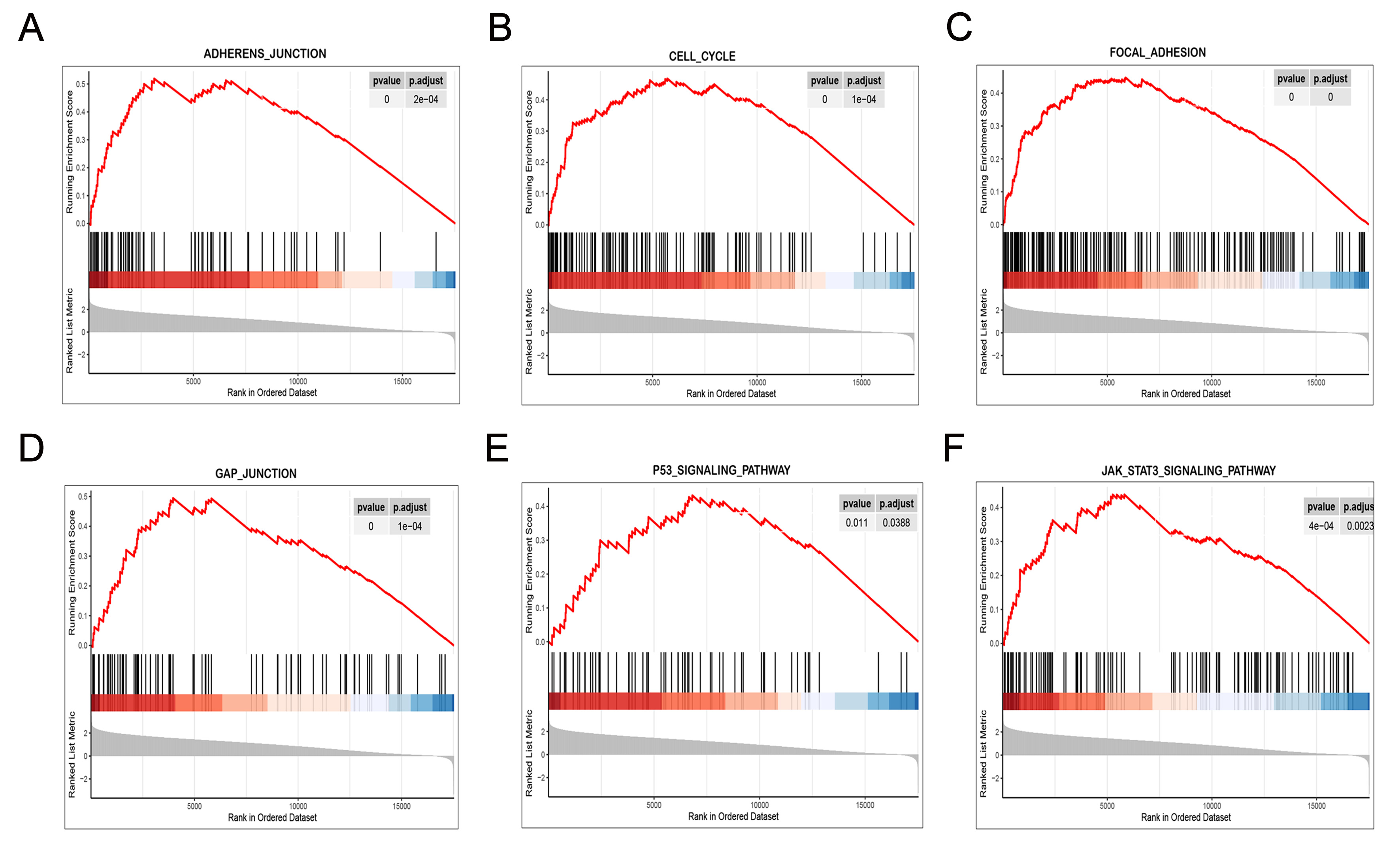


Figure S6. GSEA analysis of ZDHHC15 in glioma in the CGGA database.


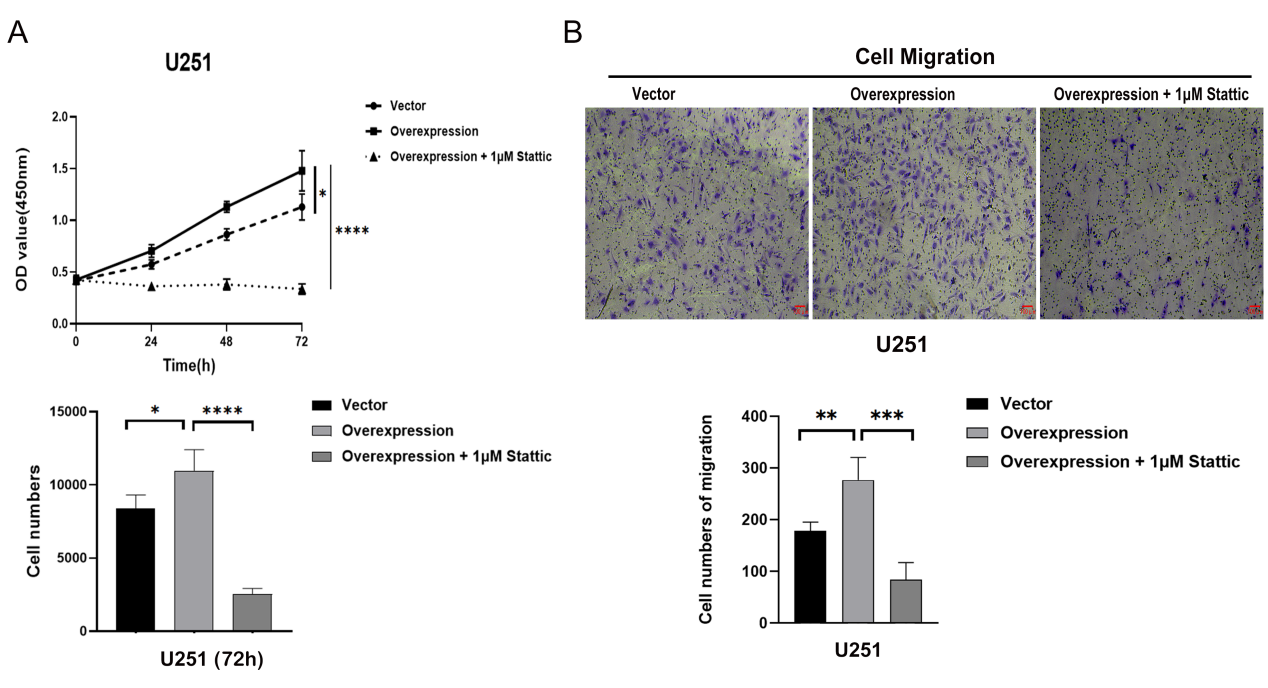


Figure S7. STAT3 inhibitor Stattic inhibits the proliferation and migration of ZDHHC15 overexpressed glioma cells. Scale bars, 100μm, Student’s t-test, ^*^*P* ＜ 0.05, ^**^*P* ＜ 0.01, ^***^*P* ＜ 0.001，^****^*P* ＜ 0.0001.

.


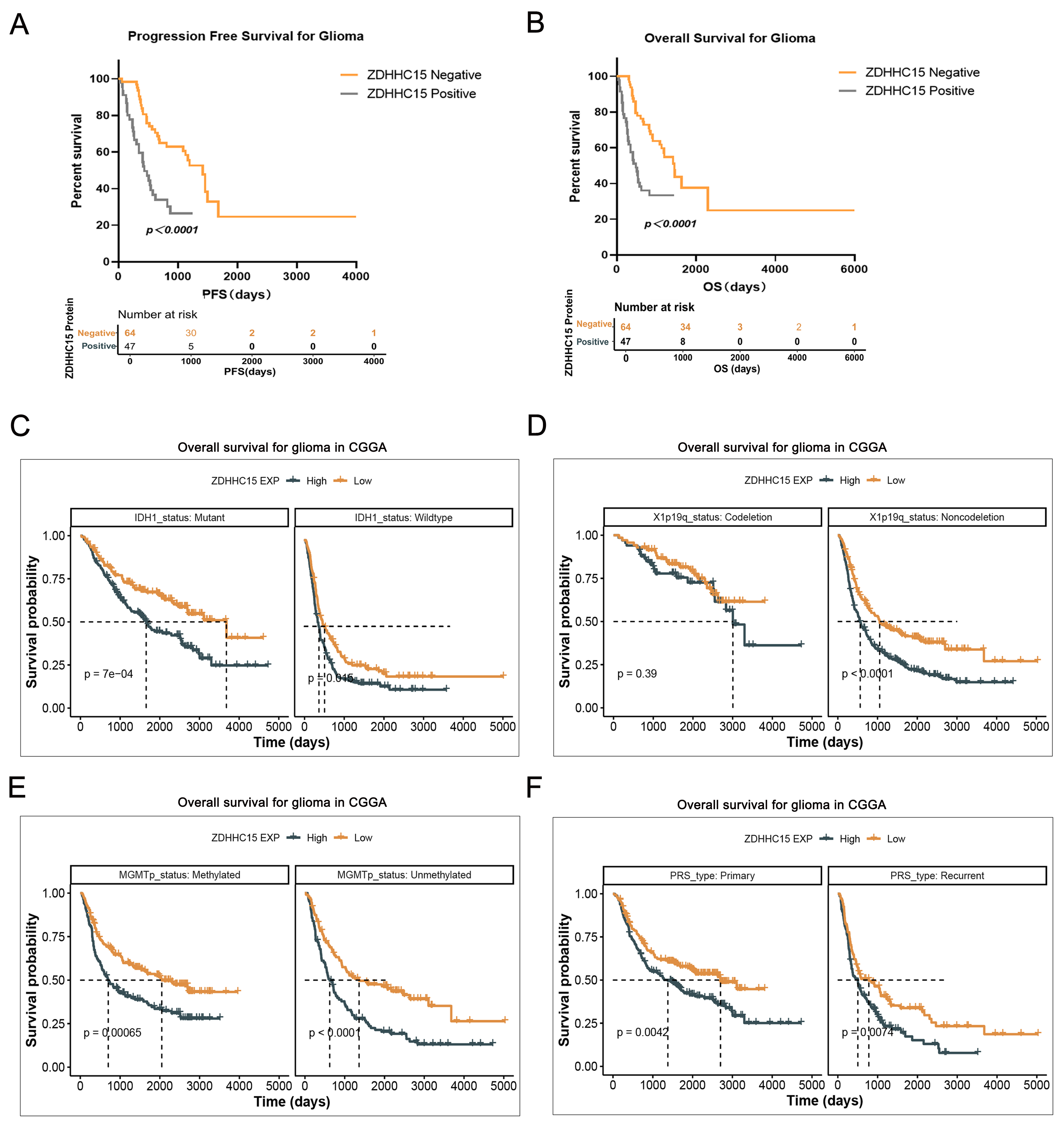


Figure S8. Prognostic values of ZDHHC15 in our cohort and CGGA database. (A, B) Progression-free survival and overall survival analysis of patients with glioma in the positive and negative ZDHHC15 protein groups in our cohort. (C-E) Kaplan-Meier survival analysis of ZDHHC15 expression in patients with different subtypes of glioma in the CGGA database.


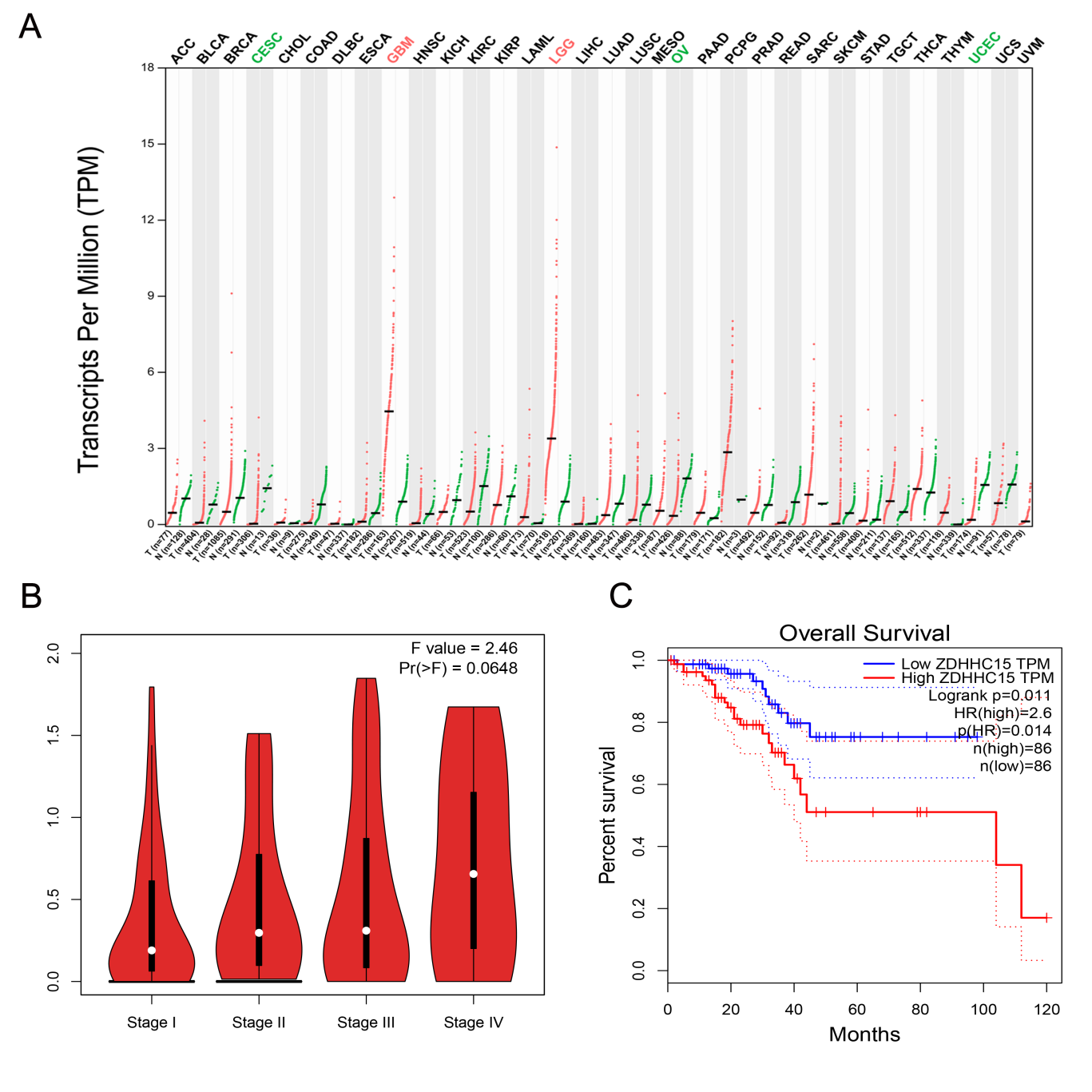


Figure S9. Expression and prognostic value of ZDHHC15 in pan-cancer analysis. (A) Relative mRNA expression levels of ZDHHC15 in all tumor types. (B) Correlation of ZDHHC15 and UCEC grades. (C) Kaplan-Meier analysis of ZDHHC15 in UCEC.

**Table S1. Clinical and pathological characteristics of 43 glioma patients.**

| Characteristics | Grade 2 | Grade 3 | Grade 4 | All patients | Pearson chi-square |
| --- | --- | --- | --- | --- | --- |
|  | n (%) | n (%) | n (%) | n (%) | P-value |
| Count | 8 (18.6) | 8 (18.6) | 27 (62.8) | 43 (100) |  |
| Gender |  |  |  |  | 0.9396 |
| Female | 4 (50.0) | 4 (50.0) | 15 (55.6) | 23 (53.5) |  |
| Male | 4 (50.0) | 4 (50.0) | 12 (44.4) | 20 (46.5) |  |
| Age (y) (Mean±SD) | 42.8±12.0 | 49.5±13.1 | 53.8±15.1 | 50.8±14.5 | 0.1087 |
| Tumor location |  |  |  |  | 0.0583 |
| Frontal | 6 (75.0) | 6 (75.0) | 9 (34.6) | 21 (50.0) |  |
| Temporal | 2 (25.0) | 0 (0) | 5 (19.2) | 7 (16.7) |  |
| Others | 0 (0) | 2 (25.0) | 12 (46.2) | 14 (33.3) |  |
| Tumor size, cm^3^ |  |  |  |  | 0.2704 |
| ＜12 | 4 (50.0) | 6 (75.0) | 11 (42.3) | 21 (50.0) |  |
| ≥12 | 4 (50.0) | 2 (25.0) | 15 (57.7) | 21 (50.0) |  |
| Symptoms at the diagnosis |  |  |  |  | 0.9283 |
| Epilepsy | 2 (25.0) | 1 (12.5) | 3 (11.5) | 6 (14.3) |  |
| Headache | 2 (25.0) | 4 (50.0) | 7 (26.9) | 13 (31.0) |  |
| Dizziness | 1 (12.5) | 1 (12.5) | 3 (11.5) | 5 (11.9) |  |
| Limb weakness | 2 (25.0) | 1 (12.5) | 7 (26.9) | 10 (23.8) |  |
| Others | 1 (12.5) | 1 (12.5) | 6 (23.2) | 8 (19.0) |  |
| Karnofsky Performance Scale |  |  |  |  | 0.0122 |
| ＜90 | 1 (12.5) | 0 (0) | 13 (50.0) | 14 (33.3) |  |
| ≥90 | 7 (87.5) | 8 (100.0) | 13 (50.0) | 28 (66.7) |  |
| Tumor recurrence |  |  |  |  | 0.1657 |
| Yes | 2(25.0) | 0 (0) | 7 (43.8) | 9 (31.0) |  |
| No | 6 (75.0) | 5 (100.0) | 9 (56.2) | 20 (69.0) |  |

**Table S2. Clinical and pathological characteristics of 143 glioma patients.**

| Characteristics | Grade 2 | Grade 3 | Grade 4 | All patients | Pearson chi-square |
| --- | --- | --- | --- | --- | --- |
|  | n (%) | n (%) | n (%) | n (%) | P-value |
| Count | 34 (23.8) | 25 (17.5) | 84 (58.7) | 143 (100) |  |
| Gender |  |  |  |  | 0.6776 |
| Female | 16 (47.1) | 9 (36.0) | 34 (40.5) | 59 (41.3) |  |
| Male | 18 (52.9) | 16 (64.0) | 50 (59.5) | 84 (58.7) |  |
| Age (y) (Mean±SD) | 42.8±9.0 | 46.3±12.1 | 54.5±12.5 | 50.3±12.5 | ＜0.0001 |
| Tumor location |  |  |  |  | 0.0305 |
| Frontal | 13 (39.4) | 13 (54.2) | 22 (27.5) | 48 (35.0) |  |
| Temporal | 8 (24.2) | 1 (4.2) | 11 (13.8) | 20 (14.6) |  |
| Others | 12 (36.4) | 10 (41.6) | 47 (58.7) | 69 (50.4) |  |
| Tumor size, cm^3^ |  |  |  |  | 0.6268 |
| ＜9.5 | 18 (54.5) | 14 (56.0) | 38 (46.9) | 70 (50.4) |  |
| ≥9.5 | 15 (45.5) | 11 (44.0) | 43 (53.1) | 69 (49.6) |  |
| Symptoms at the diagnosis |  |  |  |  | 0.0156 |
| Epilepsy | 10 (30.3) | 10 (40.0) | 6 (7.2) | 26 (18.4) |  |
| Headache | 9 (27.3) | 7 (28.0) | 31 (37.3) | 47 (33.3) |  |
| Dizziness | 4 (12.1) | 4 (16.0) | 18 (21.7) | 26 (18.5) |  |
| Limb weakness | 4 (12.1) | 2 (8.0) | 11 (13.3) | 17 (12.1) |  |
| Others | 6 (18.2) | 2 (8.0) | 17 (20.5) | 25 (17.7) |  |
| Karnofsky Performance Scale |  |  |  |  | 0.0016 |
| ＜90 | 7 (21.2) | 8 (32.0) | 46 (55.4) | 61 (43.3) |  |
| ≥90 | 26 (78.8) | 17 (68.0) | 37 (44.6) | 80 (56.7) |  |
| Radio- and/or chemotherapy |  |  |  |  | 0.8532 |
| Yes | 26 (89.7) | 19 (86.4) | 67 (90.5) | 112 (89.6) |  |
| No | 3 (10.3) | 3 (13.6) | 7 (9.5) | 13 (10.4) |  |
| Tumor recurrence |  |  |  |  | 0.3895 |
| Yes | 8 (25.0) | 3 (13.0) | 22 (26.8) | 33 (24.1) |  |
| No | 24 (75.0) | 20 (87.0) | 60 (73.2) | 104 (75.9) |  |
| ZDHHC15 intensity |  |  |  |  | 0.0050 |
| - | 32 (94.1) | 15 (60.0) | 46 (54.8) | 93 (65.0) |  |
| + | 2 (5.9) | 9 (36.0) | 28 (33.3) | 39 (27.3) |  |
| ++ | 0 (0) | 1 (4.0) | 8 (9.5) | 9 (6.3) |  |
| +++ | 0 (0) | 0 (0) | 2 (2.4) | 2 (1.4) |  |

# Original Images for Blots


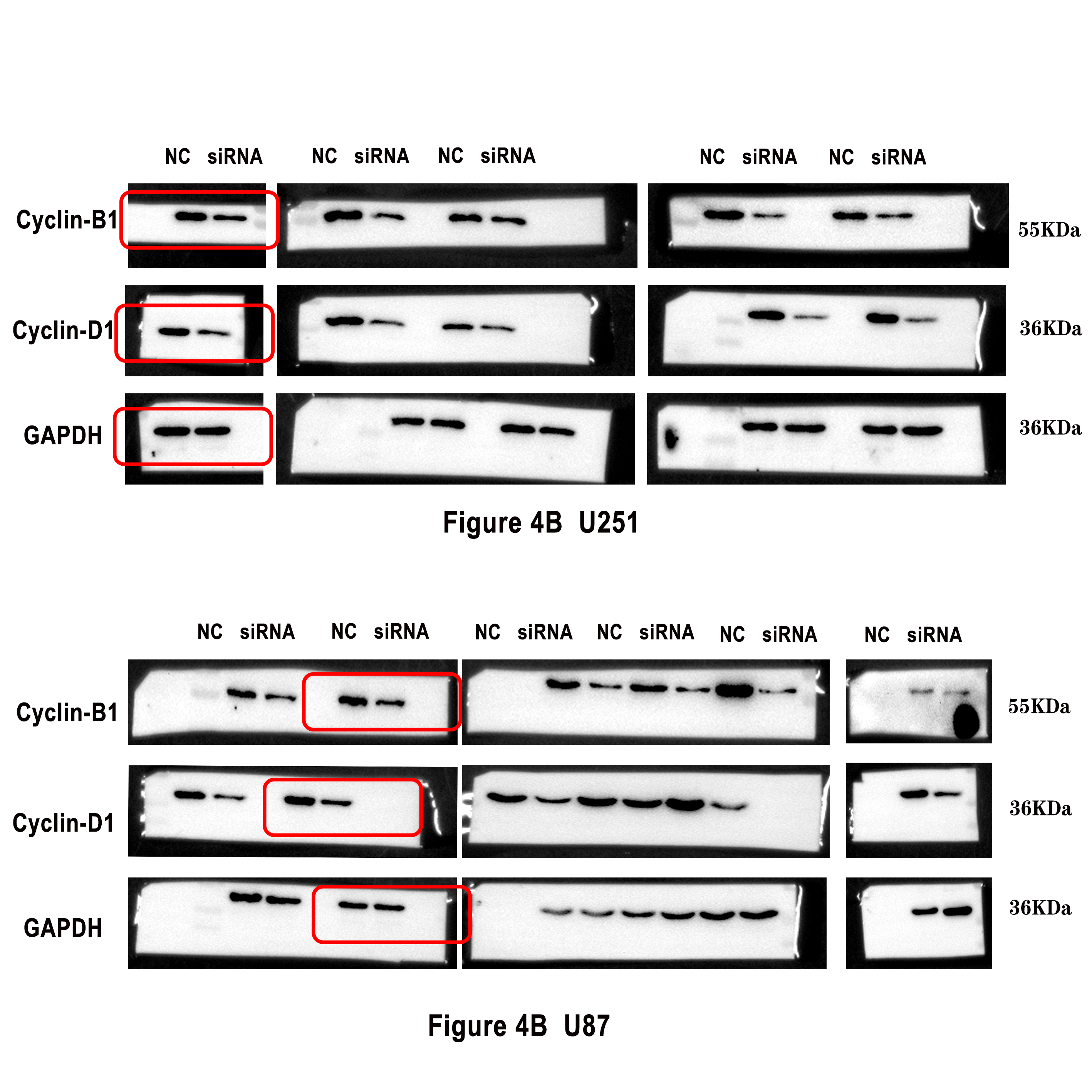


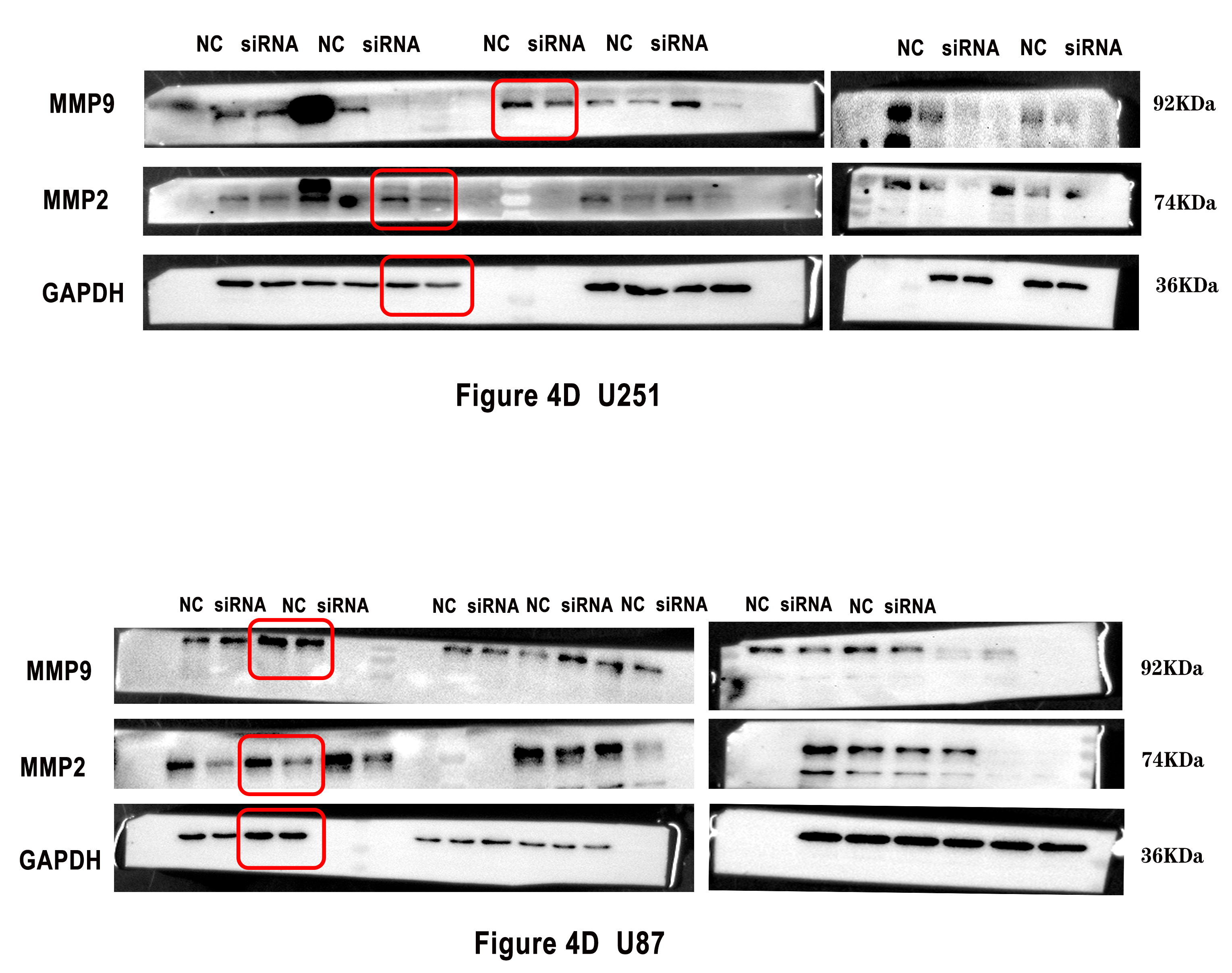


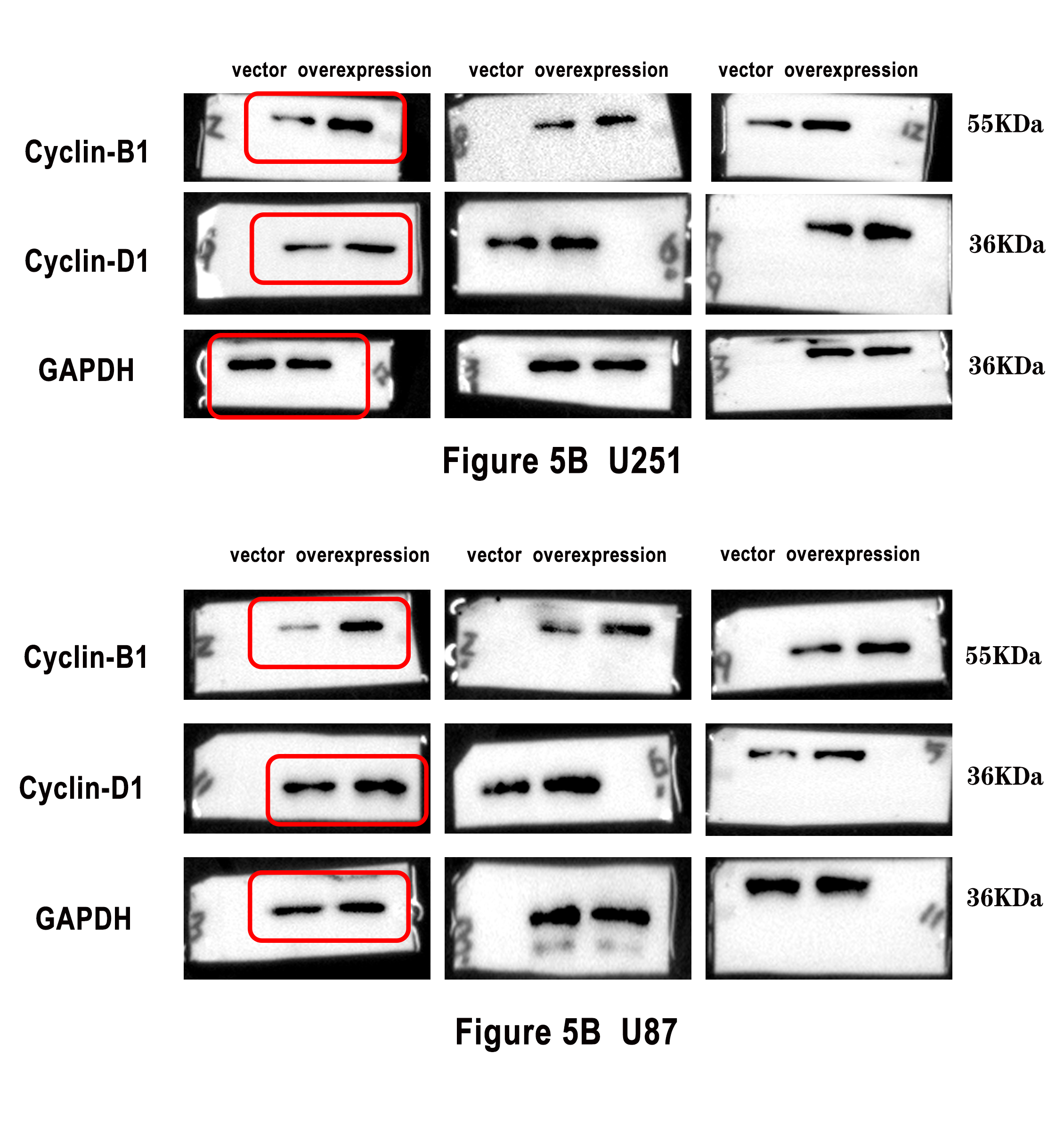


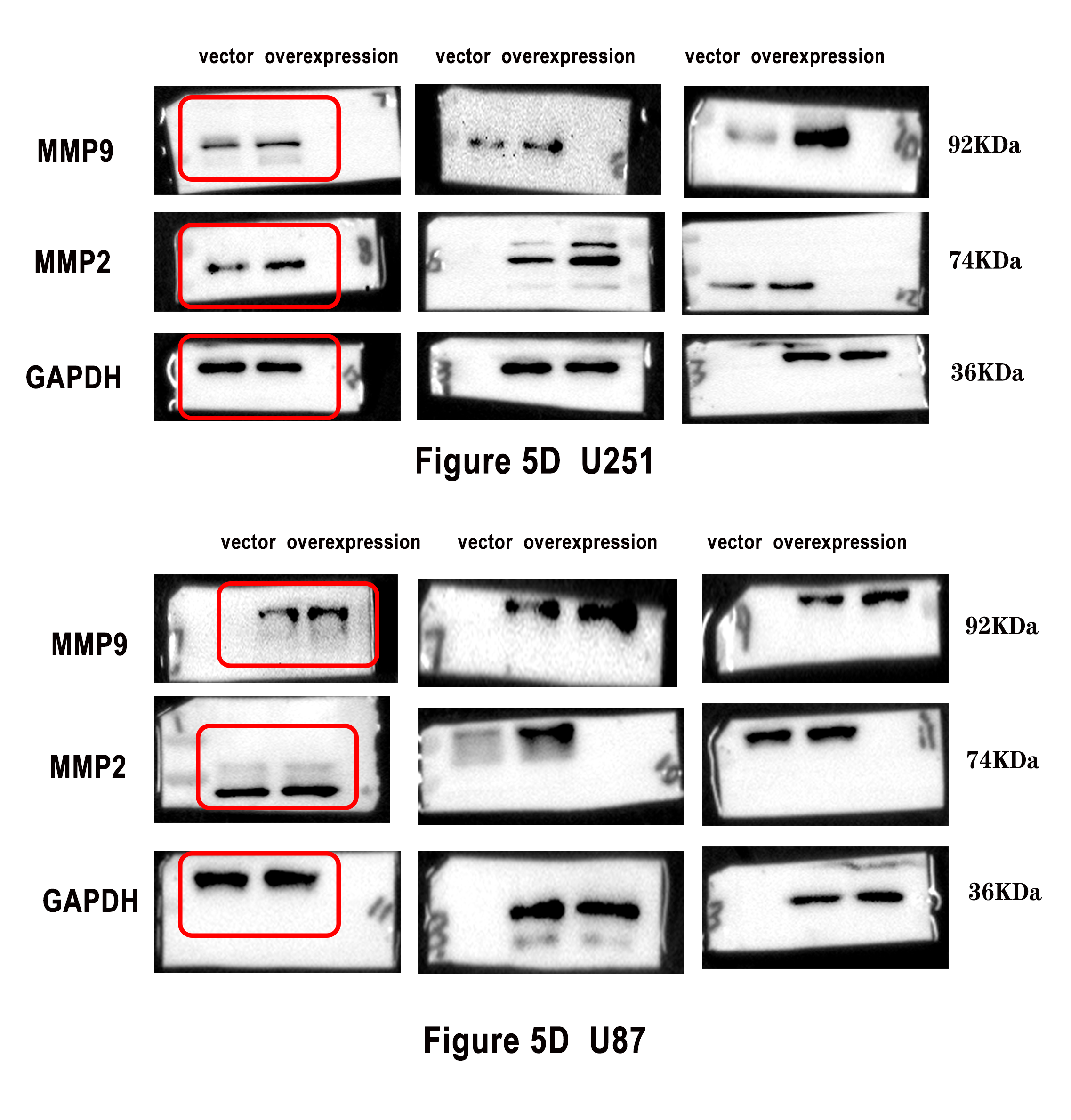


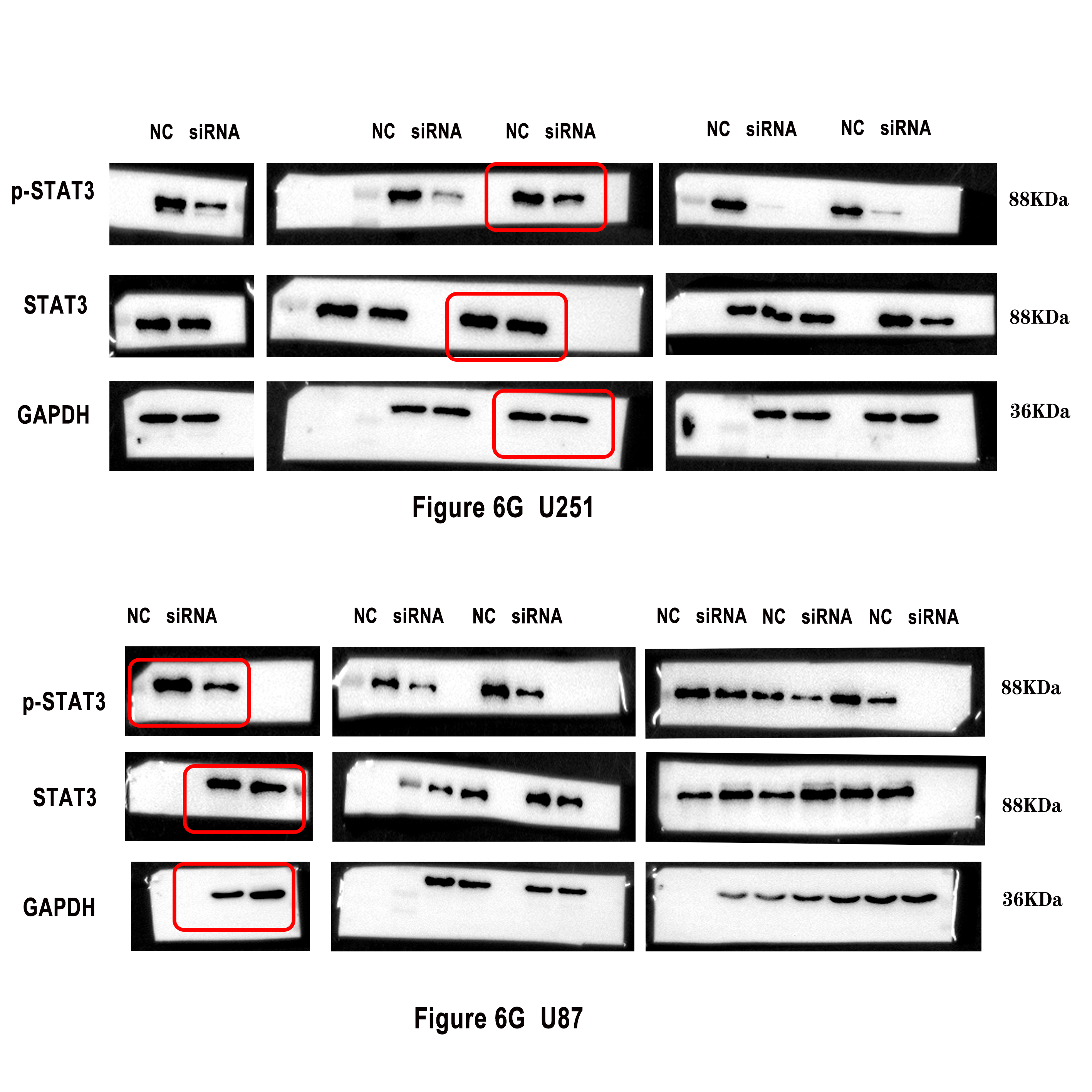


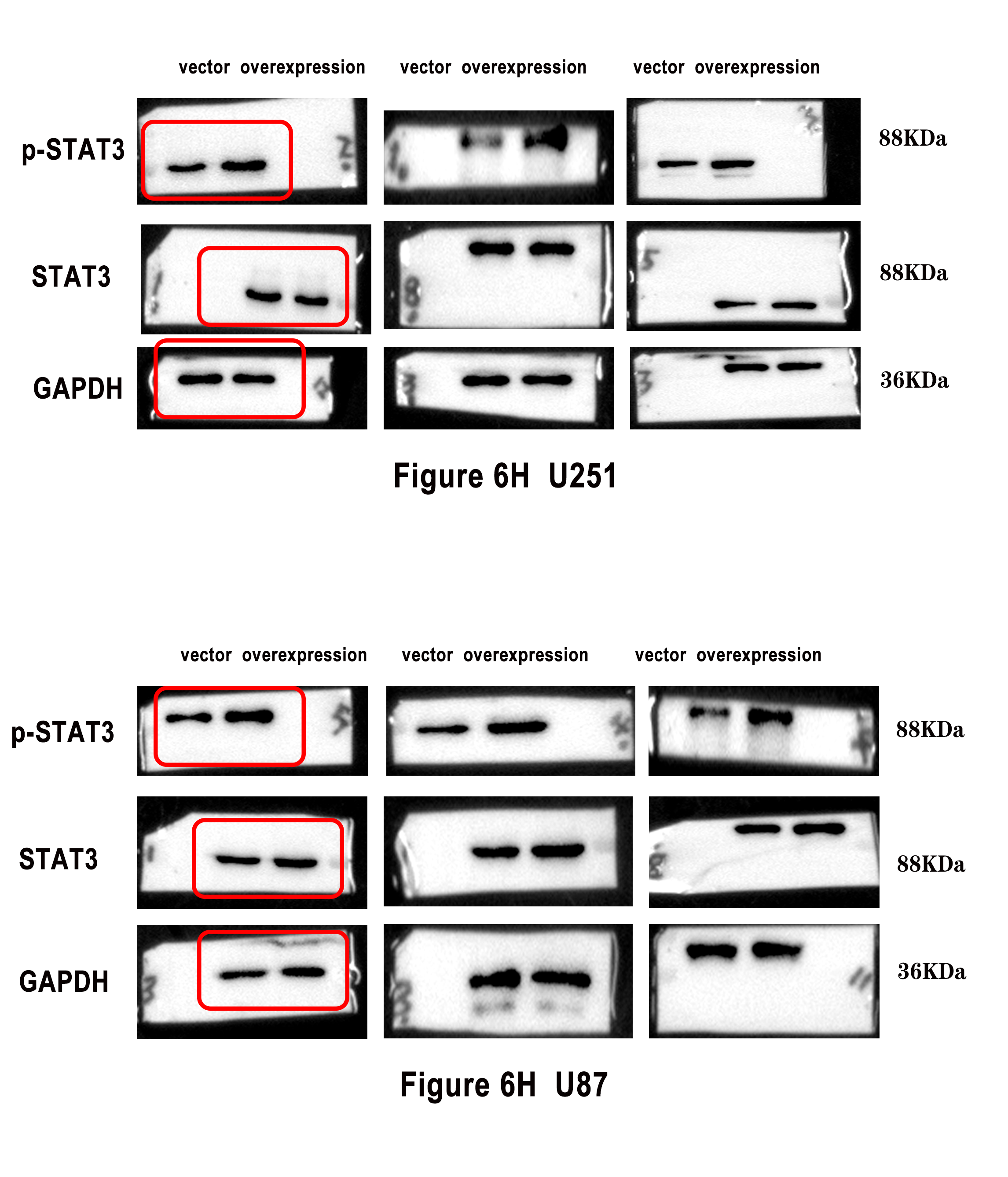


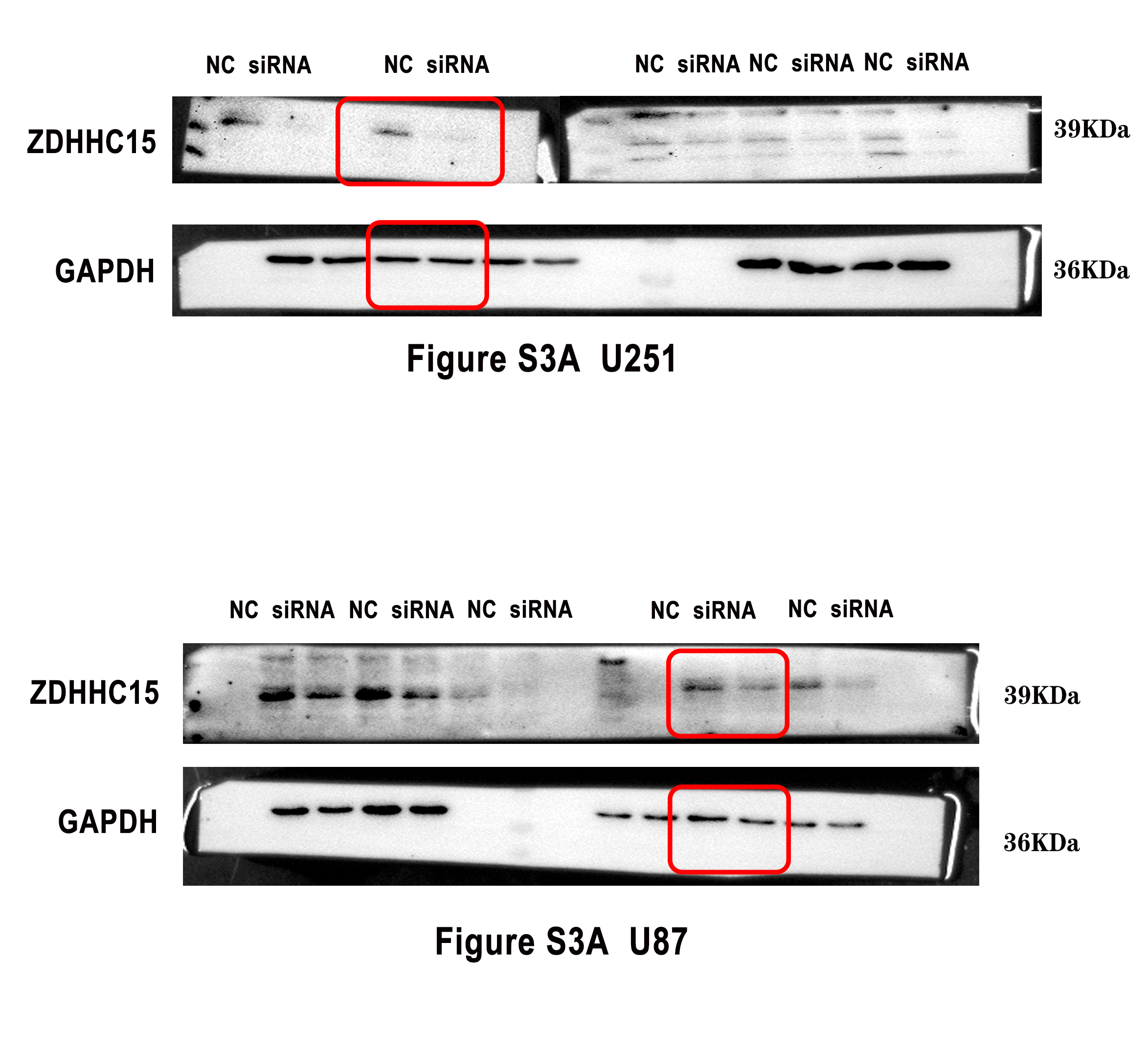

Supplement: Supplementary file 1 — Supplementary Material 1 [file 12885_2023_10883_MOESM1_ESM.docx]
